# Correction: *Wolbachia* Blocks Viral Genome Replication Early in Infection without a Transcriptional Response by the Endosymbiont or Host Small RNA Pathways

**DOI:** 10.1371/journal.ppat.1005639

**Published:** 2016-05-09

**Authors:** The PLOS Pathogens Staff

## Notice of Republication

This article was republished on April 25, 2016 to correct errors in the figures that were introduced during the typesetting process. Supplemental Figures 1–5 were published in place of Figures 1–5. The publisher apologizes for the errors. Please download this article again to view the correct version. The originally published, uncorrected article and the republished, corrected articles are provided here for reference.

## Supporting Information

S1 File Originally published, uncorrected article. (PDF)

S2 File Republished, corrected article. (PDF)
