# Supplementary figures and images for: Wolbachia Blocks Viral Genome Replication Early in Infection without a Transcriptional Response by the Endosymbiont or Host Small RNA Pathways
Source: PLoS Pathog. 2016 Apr 18;12(4):e1005536. doi: 10.1371/journal.ppat.1005536 (PMC4835223; doi:10.1371/journal.ppat.1005536)

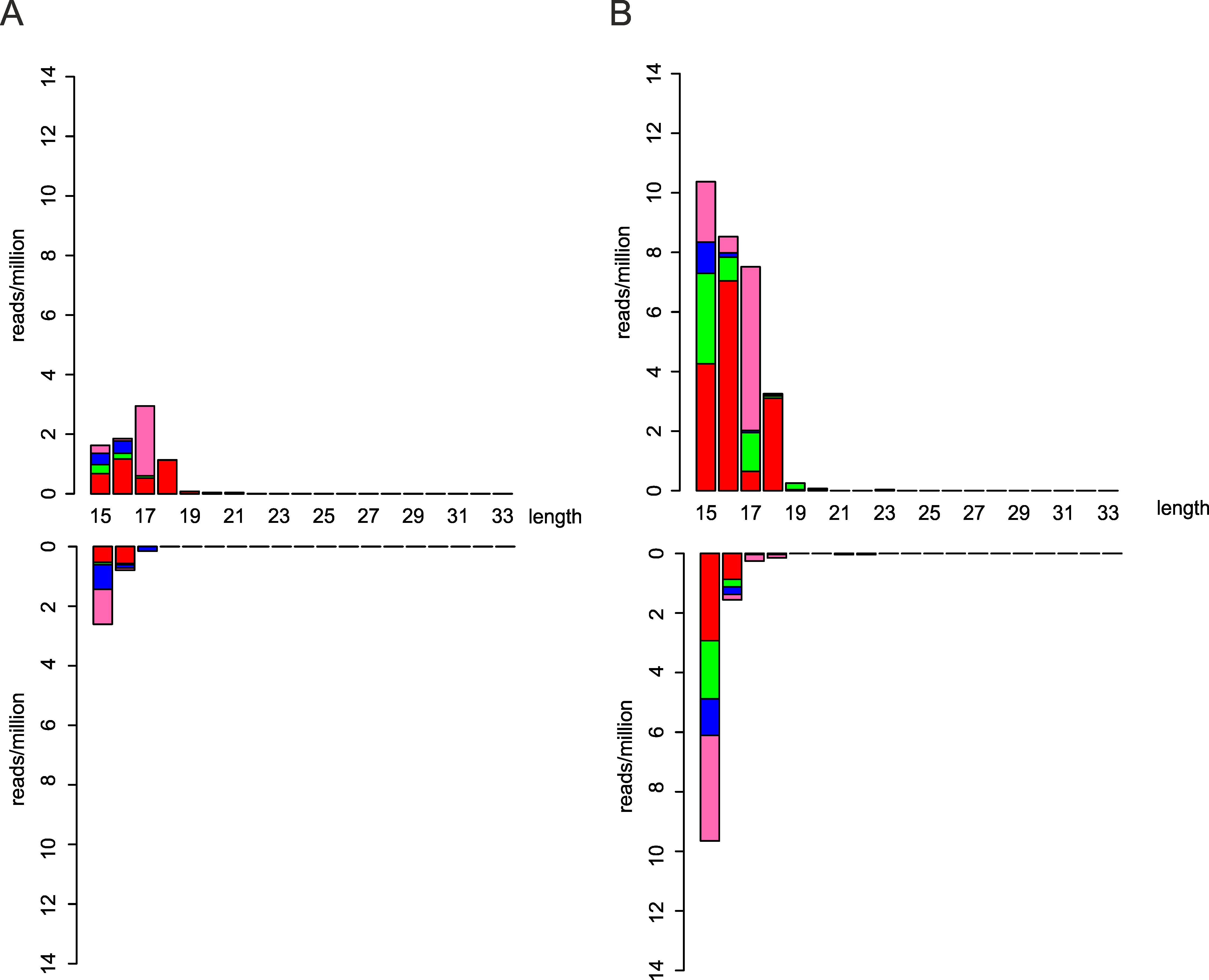

Supplement: S1 Fig — This data is the virus-free controls for Fig 4A and 4B in the main text. The length and first nucleotide distribution of small RNAs mapping to SFV genome (upper bars, 5′-3′ orientation) or antigenome (lower bars, 3’-5’ orientation) at 24 h post mock infection of D. melanogaster cells in the absence (A) (Jw18Free) or presence (B) (Jw18Wol) of Wolbachia are shown. Concatenated data from 5 independent infections are shown. A = red, C = green, G = blue and T = pink. (TIF) [file ppat.1005536.s001.tif]

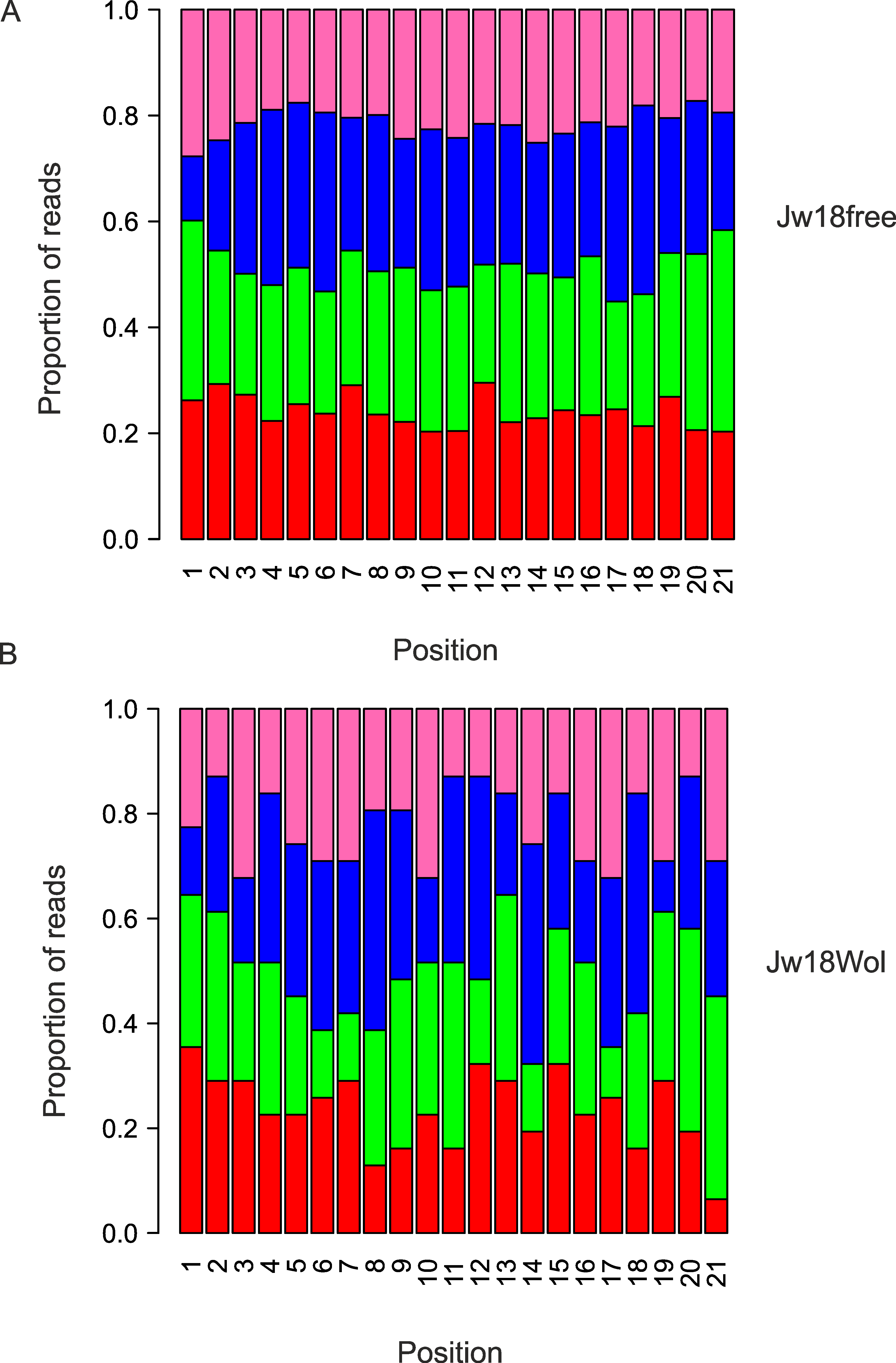

Supplement: S2 Fig — Mapping of nucleotide distribution of small RNAs mapping to the SFV genome in the (A) (Jw18Free) absence or (B) (Jw18Wol) presence of Wolbachia. A = red, C = green, G = blue and T = pink. (TIF) [file ppat.1005536.s002.tif]

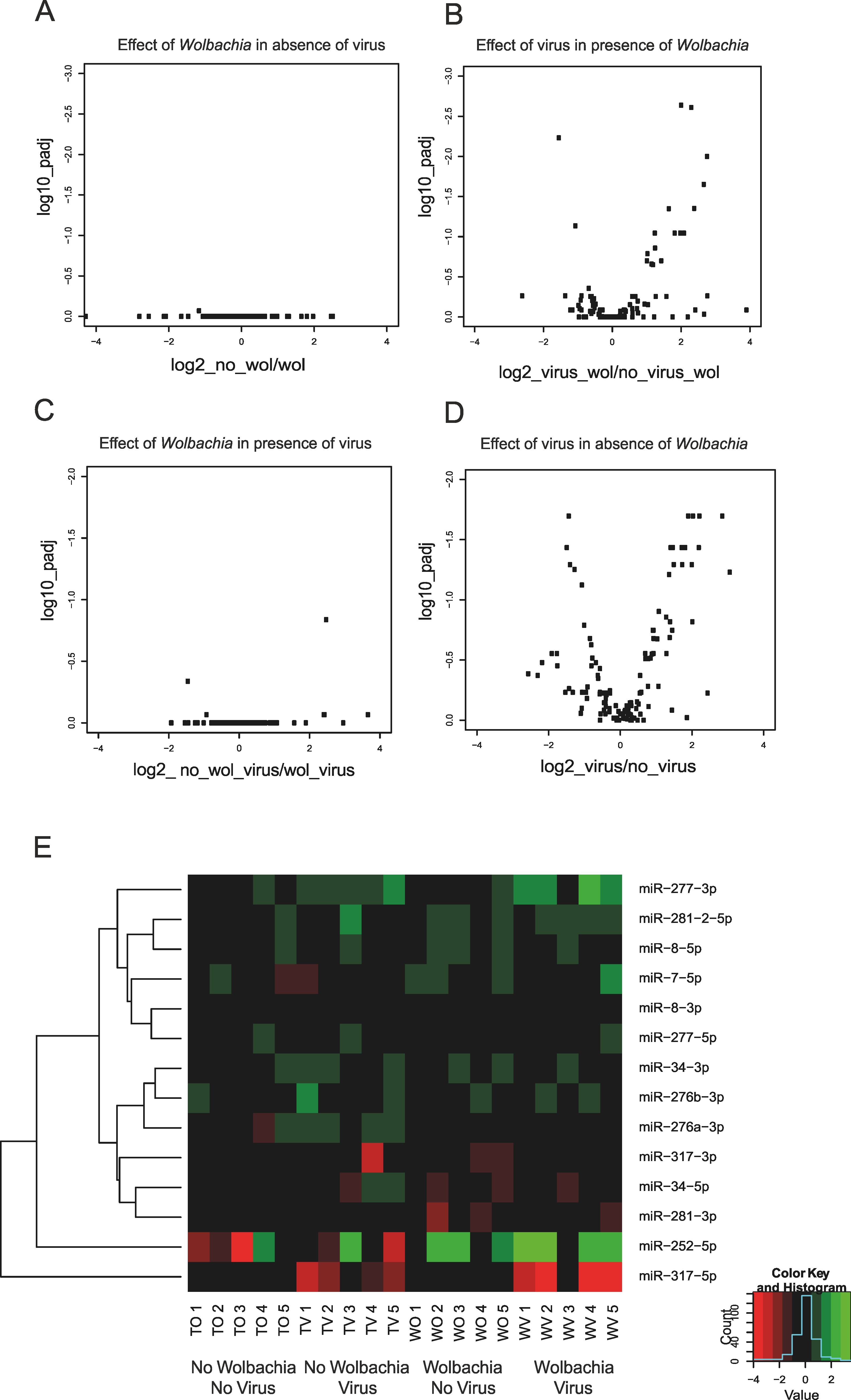

Supplement: S3 Fig — Panels (A-D) are volcano plots summarizing the differential expression of miRNAs between pairs of treatments. The Y axis is the log10 of the FDR corrected P value. The X axis is the change in expression on a log2 scale. (E) Heatmap showing the relative expression of miRNAs homologous to those reported to be affected by Wolbachia infection of mosquito cells [48]. T = Jw18Free, W = Jw18Wol, V = SFV infection, O = no SFV infection and numbers represent replicate. (TIF) [file ppat.1005536.s003.tif]

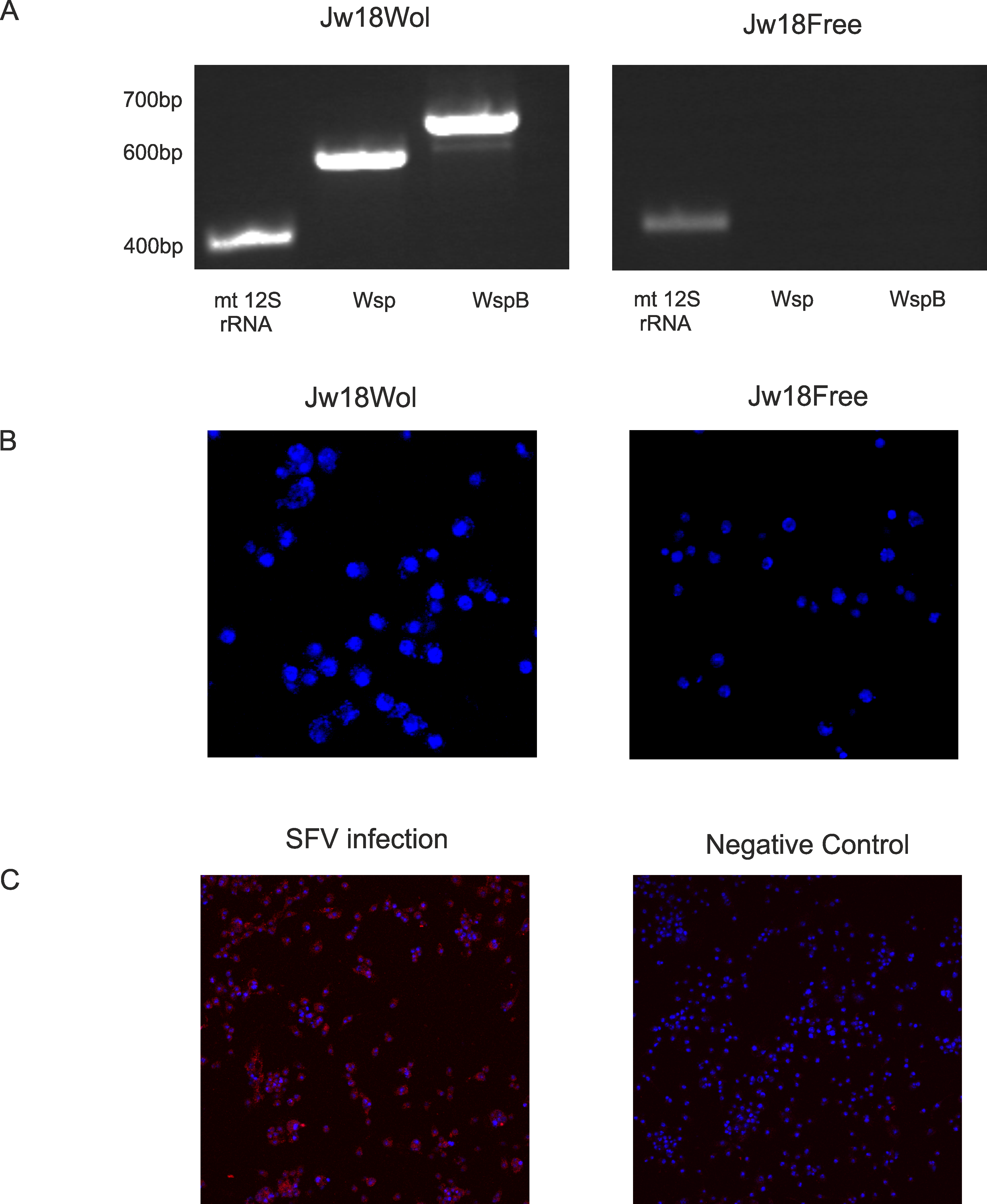

Supplement: S4 Fig — Cells were tetracycline treated or not and stained with DAPI in order to visualize Wolbachia infection; tetracycline-treated cells are referred to as Jw18Free. (A) Cells were checked for the presence of Wolbachia by PCR using two separate primer pairs as described previously [6]. (B) Cells were then stained with DAPI in order to visualise Wolbachia content, density was consistent with previous observations with ~90% of cells infected. (C) Jw18Free cells were infected with an MOI of 20 calculated in BHK cells and stained with SFV NSP2 antibody [50] in order to determine infection rate. Cells positive for SFV were shown to have an infection rate >90%. (TIF) [file ppat.1005536.s004.tif]

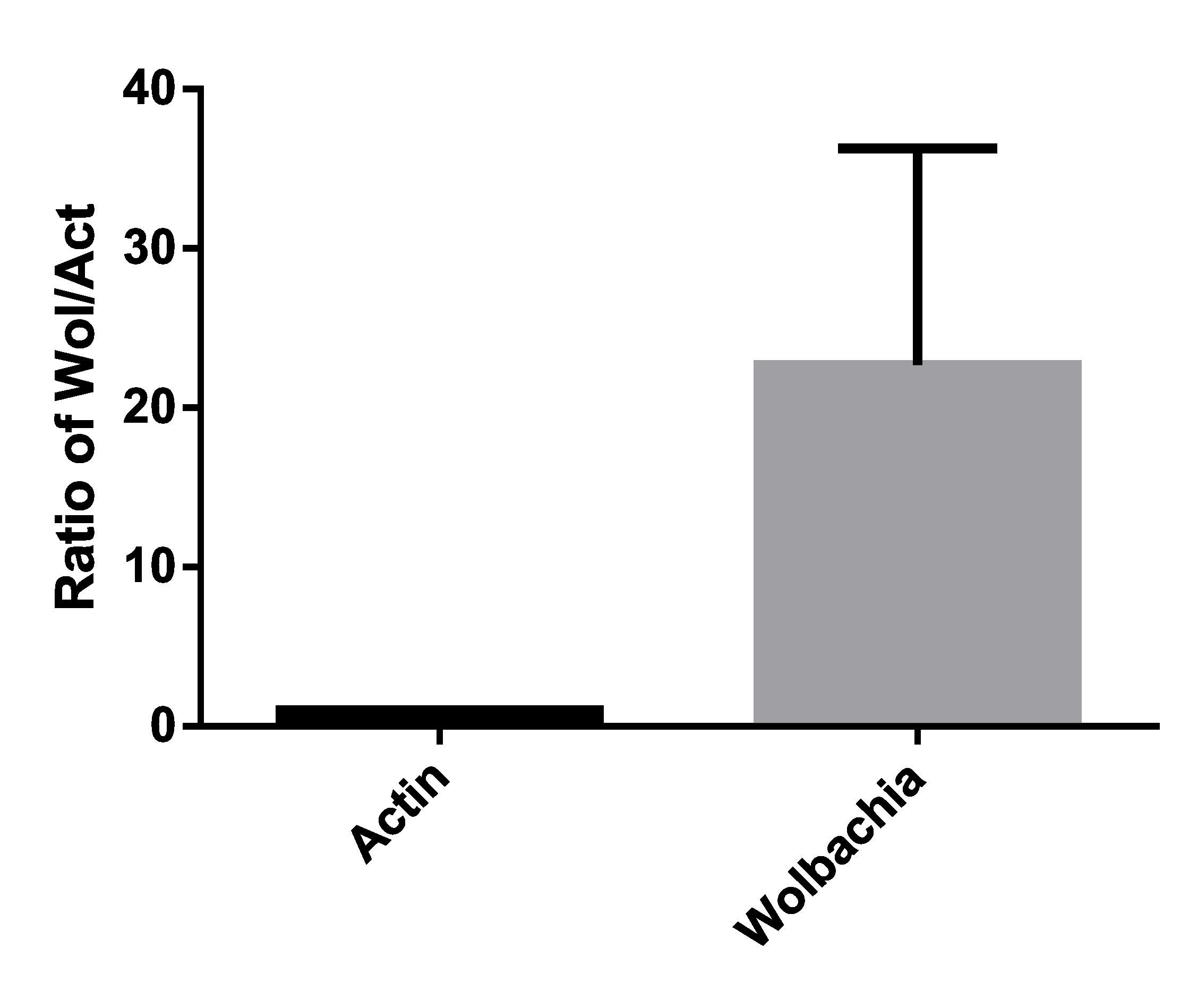

Supplement: S5 Fig — Wolbachia density was calculated as a ratio to the endogenous control Actin 5C (Wol/Act), where it is assumed there is one copy of actin per cell. Experiments were carried out in triplicate with two biological replicates. Error bar indicates standard deviation. (TIF) [file ppat.1005536.s005.tif]
