# Supplementary material for: Wolbachia Blocks Viral Genome Replication Early in Infection without a Transcriptional Response by the Endosymbiont or Host Small RNA Pathways
Source: PLoS Pathog. 2016 Apr 18;12(4):e1005536. doi: 10.1371/journal.ppat.1005536 (PMC4835223; doi:10.1371/journal.ppat.1005536)
Supplement: S1 Table — Table indicates miRNAs found to be significantly differentially expressed (Adjusted p value<0.1, Negative Binomial Test) upon SFV infection in either cells positive (Jw18Wol) or negative (Jw18Free) for Wolbachia. Black means significantly upregulated and red significantly down regulated. (DOCX) [file ppat.1005536.s006.docx]

|  | MicroRNA | log2 fold  Virus over no virus |
| --- | --- | --- |
| Jw18Wol cells |  | |
|  | miR-304-5p | 3.517295834 |
|  | miR-282-3p | 3.418987722 |
|  | miR-79-3p | 2.775395147 |
|  | miR-983-5p | 2.679525517 |
|  | miR-996-3p | 2.401896614 |
|  | miR-304-3p | 2.3178206 |
|  | miR-1003-3p | 2.105781565 |
|  | miR-283-5p | 2.023631507 |
|  | miR-308-5p | 1.998564786 |
|  | miR-137-5p | 1.834651565 |
|  | miR-996-5p | 1.664647319 |
|  | miR-277-3p | 1.257867187 |
|  | miR-279-3p | -1.052530737 |
|  | miR-7-5p | -1.533116501 |
|  | miR-317-5p | -1.606611814 |
| Jw18Free cells |  | |
|  | miR-1006-3p | 3.075970438 |
|  | miR-1003-3p | 2.866635126 |
|  | miR-315-5p | 2.229454545 |
|  | miR-79-3p | 2.213025475 |
|  | miR-184-5p | 2.04976124 |
|  | miR-967-5p | 2.017279957 |
|  | miR-283-5p | 1.917312413 |
|  | miR-304-5p | 1.831733841 |
|  | miR-996-5p | 1.817449453 |
|  | miR-282-3p | 1.749954698 |
|  | miR-304-3p | 1.735844526 |
|  | miR-13b-3p | 1.514999315 |
|  | miR-306-5p | 1.485081462 |
|  | miR-11-5p | 1.456720494 |
|  | miR-33-3p | 1.426871727 |
|  | miR-1013-3p | 1.384144736 |
|  | miR-286-3p | -1.048957574 |
|  | miR-998-3p | -1.253807551 |
|  | miR-7-5p | -1.380295556 |
|  | miR-317-5p | -1.416271974 |
|  | miR-279-3p | -1.471980489 |
