# Supplementary material for: Correction: Wolbachia Blocks Viral Genome Replication Early in Infection without a Transcriptional Response by the Endosymbiont or Host Small RNA Pathways
Source: PLoS Pathog. 2016 May 9;12(5):e1005639. doi: 10.1371/journal.ppat.1005639 (PMC4861256; doi:10.1371/journal.ppat.1005639)
Supplement: S1 File — (PDF) [file ppat.1005639.s001.PDF]

PLOS | PATHOGENS

RESEARCH ARTICLE

# *Wolbachia* Blocks Viral Genome Replication Early in Infection without a Transcriptional Response by the Endosymbiont or Host Small RNA Pathways

**Stephanie M. Rainey[1☯], Julien Martinez[2☯], Melanie McFarlane[1], Punita Juneja[2], Peter Sarkies[3], Aleksei Lulla[4], Esther Schnettler[1], Margus Varjak[1], Andres Merits[4], Eric A. Miska[5], Francis M. Jiggins[2]*, Alain Kohl[1]***

**1** MRC-University of Glasgow Centre for Virus Research, Glasgow, Scotland, United Kingdom, **2** Department of Genetics, University of Cambridge, Cambridge, United Kingdom, **3** MRC Clinical Sciences Centre, Imperial College London, London, United Kingdom, **4** Institute of Technology, University of Tartu, Tartu, Estonia, **5** Gurdon Institute and Department of Genetics, University of Cambridge, Cambridge, United Kingdom

☯ These authors contributed equally to this work.
* alain.kohl@glasgow.ac.uk (AK); fmj1001@cam.ac.uk (FMJ)

OPEN ACCESS

**Citation:** Rainey SM, Martinez J, McFarlane M, Juneja P, Sarkies P, Lulla A, et al. (2016) *Wolbachia* Blocks Viral Genome Replication Early in Infection without a Transcriptional Response by the Endosymbiont or Host Small RNA Pathways. PLoS Pathog 12(4): e1005536. doi:10.1371/journal.ppat.1005536

**Editor:** Elizabeth Ann McGraw, Monash University, AUSTRALIA

**Received:** November 10, 2015

**Accepted:** March 9, 2016

**Published:** April 18, 2016

**Copyright:** © 2016 Rainey et al. This is an open access article distributed under the terms of the Creative Commons Attribution License, which permits unrestricted use, distribution, and reproduction in any medium, provided the original author and source are credited.

**Data Availability Statement:** All sequencing files are available from the Sequence Read Archive database (total RNA, accession number PRJEB10681; small RNA, accession number PRJEB9710).

**Funding:** This study was funded by the Wellcome Trust grant WT094664MA (http://www.wellcome.ac.uk/) (AK, FMJ). FMJ is also supported by a Royal Society Research Fellowship (https://royalsociety.org). AK and ES are supported by the MRC grant MC_UU_12014 (www.mrc.ac.uk). We also acknowledge Wellcome Trust and Cancer Research

## Abstract

The intracellular endosymbiotic bacterium *Wolbachia* can protect insects against viral infection, and is being introduced into mosquito populations in the wild to block the transmission of arboviruses that infect humans and are a major public health concern. To investigate the mechanisms underlying this antiviral protection, we have developed a new model system combining *Wolbachia*-infected *Drosophila melanogaster* cell culture with the model mosquito-borne Semliki Forest virus (SFV; *Togaviridae*, *Alphavirus*). *Wolbachia* provides strong antiviral protection rapidly after infection, suggesting that an early stage post-infection is being blocked. *Wolbachia* does appear to have major effects on events distinct from entry, assembly or exit as it inhibits the replication of an SFV replicon transfected into the cells. Furthermore, it causes a far greater reduction in the expression of proteins from the 3´ open reading frame than the 5´ non-structural protein open reading frame, indicating that it is blocking the replication of viral RNA. Further to this separation of the replicase proteins and viral RNA in transreplication assays shows that uncoupling of viral RNA and replicase proteins does not overcome *Wolbachia's* antiviral activity. This further suggests that replicative processes are disrupted, such as translation or replication, by *Wolbachia* infection. This may occur by *Wolbachia* mounting an active antiviral response, but the virus did not cause any transcriptional response by the bacterium, suggesting that this is not the case. Host microRNAs (miRNAs) have been implicated in protection, but again we found that host cell miRNA expression was unaffected by the bacterium and neither do our findings suggest any involvement of the antiviral siRNA pathway. We conclude that *Wolbachia* may directly interfere with early events in virus replication such as translation of incoming viral RNA or RNA transcription, and this likely involves an intrinsic (as opposed to an induced) mechanism.

UK (www.cancerresearchuk.org) core support and a Wellcome Trust Senior Investigator Award (EAM). The funders had no role in study design, data collection and analysis, decision to publish, or preparation of the manuscript.

**Competing Interests:** The authors have declared that no competing interests exist.

## Author Summary

The intracellular endosymbiotic bacterium *Wolbachia* can protect insects against viral infection. However, the mechanisms underlying this antiviral activity are poorly understood. We have developed a new model system combining *Wolbachia*-infected *Drosophila melanogaster* cell culture and the model mosquito-borne virus, Semliki Forest virus. *Wolbachia* confers strong antiviral activity against SFV. Our study indicates that viral replication appears to be inhibited at a very early stage, such as initial translation or replication. Results indicate that *Wolbachia* does not mount a transcriptional response to SFV infection and that host small RNA pathways are not involved in *Wolbachia* mediated antiviral activity in our system. We conclude that *Wolbachia* may directly interfere with early events in virus replication such as translation of incoming viral RNA or RNA transcription, and this likely involves an intrinsic (as opposed to an induced) mechanism.

## Introduction

Arthropod-borne viruses (arboviruses) pose a considerable threat to human and animal health, yet effective control measures have proven difficult to implement [1, 2]. In recent years novel means of reducing their replication in arthropod vectors have been suggested as an alternative way to reduce the prevalence of these viruses. One of the most exciting approaches is the use of the endosymbiotic intracellular bacterium *Wolbachia* to control arbovirus transmission from mosquito to vertebrate from within the arthropod vector [3, 4]. *Wolbachia* was first found to confer resistance to viruses in *Drosophila melanogaster* [5, 6]. When it was transferred to the mosquito *Aedes aegypti* it made the mosquitoes resistant to two important human pathogenic arboviruses, dengue virus (DENV) and chikungunya virus (CHIKV) [7, 8]. Importantly, *Wolbachia* can also invade and be stably maintained in natural populations thanks to a trait called cytoplasmic incompatibility, which causes embryos to die when uninfected females mate with infected males [9]. This allows *Wolbachia* to spread through mosquito populations by providing a reproductive advantage to the *Wolbachia*-infected females that transmit the bacterium [10]. Field trials have shown that releasing *Wolbachia*-infected mosquitoes allows the bacterium to invade *Ae. aegypti* populations [11, 12] and reduces the susceptibility of the mosquitoes to DENV [13].

The mechanism(s) by which *Wolbachia* confers broad resistance remains unclear. Antiviral protection is seen in insects that harbour high densities of *Wolbachia* [14, 15]. For example Martinez et al (2014) showed a clear correspondence between *Wolbachia* density and the level of protection against the insect viruses, *Drosophila* C virus (DCV) and Flock House virus (FHV) [16]. This phenomenon is also seen in the mosquito *Ae. albopictus*, where the endogenous *Wolbachia* strains *w*AlbA and *w*AlbB have a relatively low density especially in key tissues such as the midgut and offer little protection against DENV [17, 18]. It has also been hypothesised that *Wolbachia* protection is dependent on target cells and tissues harbouring *Wolbachia* [8, 14, 17]. Indeed, there is little evidence of *Wolbachia* and virus being present together in the same cell when either is present in a high density, suggesting that antiviral protection is cell autonomous [8, 19]. It may be a case of competition for space or cellular resources [8]. Viruses and *Wolbachia* depend on host lipids, and in *D. melanogaster* it has been shown that enriching dietary cholesterol reduced the extent to which *Wolbachia* protects against DCV [20]. It has also been suggested that there is competition for iron resources within cells, as *Wolbachia* upregulates transferrin in mosquitoes while DENV and CHIKV are thought to cause its downregulation [21, 22].

Viral replication is controlled by innate immune responses in both *D*. *melanogaster* and mosquitoes and several experiments suggested that the upregulation of immune pathways—immune-priming—may be important for *Wolbachia*-mediated antiviral activity [17, 23, 24]. However, this appears to only be the case in mosquito populations that have been transinfected with *Wolbachia* strains [21, 24]. *Drosophila* species that are naturally infected with *Wolbachia* do not show an immune-priming phenotype, yet still confer antiviral activity [6, 15, 21, 25]. RNAi is considered the most important antiviral response in insects, with double stranded viral RNA (dsRNA) being processed into short RNAs by the small interfering RNA (siRNA) pathway and directing the destruction of viral RNA [26, 27]. However, several studies have shown that *Wolbachia* provides protection in mutant *Drosophila* and cells that lack components of this pathway, ruling out a role for the siRNA pathway in *Wolbachia*-mediated protection [19, 28, 29]. There is however data that suggest the miRNA pathway may play a role in *Wolbachia* mediated protection [30–32]. *Wolbachia* has been shown to alter the expression of multiple miRNAs in mosquitoes [31]. The miRNA pathway is involved in many cellular processes, and miRNAs are produced from genome-encoded nuclear precursor RNAs that are processed into 22 nucleotide (nt) molecules that can induce target RNA degradation or inhibition of translation [26, 33].

In summary, the mechanism(s) by which *Wolbachia* confers antiviral activity are still unclear, and very little is known about exactly how the viral replication cycle is affected. Furthermore, it is not clear if *Wolbachia* itself responds to viral infection. In order to address these questions and to understand how *Wolbachia* interacts with viruses we have combined two powerful and well-studied model systems–the mosquito-borne alphavirus Semliki Forest virus (SFV; *Togaviridae*, genus *Alphavirus*) and a *Wolbachia*-containing *D*. *melanogaster* cell line [34]–and show that the endosymbiont provides strong protection against infection in this system. To identify the stage of the viral replication cycle that is likely being affected we compared SFV, SFV replicon and a SFV-based transreplicase system. We then used high-throughput sequencing to unravel the role of host small RNA pathways and the *Wolbachia* transcriptional response in antiviral protection. We find that *Wolbachia* targets the virus rapidly after infection, and is likely blocking early events in the replication of viral RNA (for example translation of incoming RNA, the switch from translation to replication or RNA transcription) within cells, though we cannot rule out an effect on entry or exit. These effects are neither associated with a host small RNA response nor a transcriptional response by the endosymbiont, but mediated by intrinsic activities.

## Results

### *Wolbachia* has no effect on cell growth but significantly decreases SFV replication in *D*. *melanogaster* Jw18 cells

We developed a model arbovirus infection system based on SFV, for which excellent molecular tools including replicons and recombinant viruses are available and which we have used extensively to study arbovirus-arthropod interactions [35–37], and the *D*. *melanogaster*-derived Jw18 cell line infected with the *Wolbachia* strain wMel [34].

As SFV does not naturally infect *D*. *melanogaster*, we first established if this virus is able to infect and replicate in Jw18 cells. We used SFV4(3H)-*RLuc*, which expresses *Renilla* luciferase, *RLuc*, from the non-structural open reading frame (Fig 1A) and has been used previously to study antiviral mechanisms in mosquito cells [36, 37].

The cells were first antibiotic treated to generate a *Wolbachia*-free control cell line, which we refer to as Jw18Free cells. These were infected at a multiplicity of infection (MOI) of 20 and cells were lysed 4, 8, 12 and 24 hours post infection (hpi) and *RLuc* activity measured. Over a

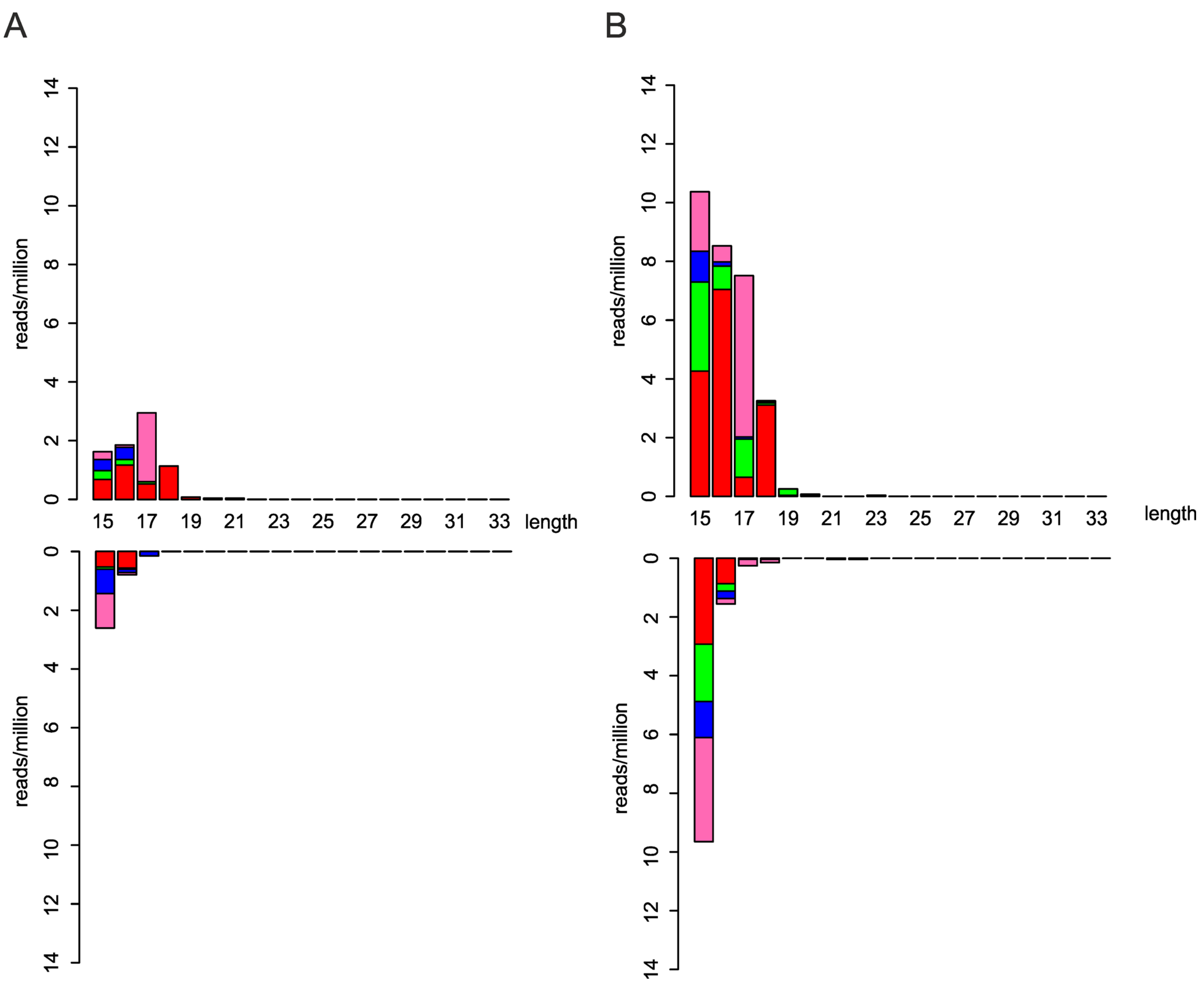


**Fig 1. Virus, replicon and transreplicase systems used in this study.** (A) Schematic representation of genome of SFV4(3H)-*RLuc*, carrying the *RLuc* reporter gene flanked by duplicated nsP2-protease cleavage sites at the nsP3/4 junction. Note that the genome is split into two major ORFs, 1 and 2, encoding non-structural and structural proteins respectively. (B) Schematic representation of the genome of viral replicon pSFV1(3F)*RLuc*-SG-*FFLuc*, where *RLuc* is fused to the region encoding for nsP3 and the structural genes have been replaced by the reporter gene firefly luciferase (*FFLuc)*. Expression of *FFLuc* occurs only from subgenomic RNA produced from the subgenomic promoter; hence detection of this marker is dependent on the active replication of transfected RNA. (C-D) Schematic representation of the SFV-derived transreplicase constructs used in this study. Expression of the replicase proteins is under the control of the *Drosophila* Actin promoter (C). Expression of SFV template RNA is also under the control of the Actin promoter (D). When the replicase proteins are expressed this leads to active replication of the template RNA. *FFLuc* expression is therefore under both the control of the Actin promoter and the SFV genomic promoter. Whereas *Gluc* is exclusively under the control of the subgenomic promoter and therefore requires active replication of the template RNA in order for expression to occur. Two replicase constructs were used in this study: one functional, and one non-functional due to the insertion of a GDD-GAA mutation in nsP4 as indicated in (C).

doi:10.1371/journal.ppat.1005536.g001

24 hour period *RLuc* activity gradually increased, indicating that SFV4(3H)-*RLuc* can infect and replicate in Jw18Free cells (Fig 2A). In order to rule out any effect of *Wolbachia* or SFV on cell growth we next compared the growth of cells Jw18Free and Jw18Wol cells either infected or not infected with SFV4(3H)-*RLuc*. There was no significant difference between any of the

treatments observed, indicating that neither *Wolbachia* nor SFV4(3H)-*RLuc* infection affected cell growth (Fig 2B). It is important to note that SFV does not cause cytopathic effects in insect cells and therefore cells are able to continue to grow even under high infection rates.

To determine if *Wolbachia* could protect against SFV infection in Jw18 cells, we infected Jw18Wol and Jw18Free cells with SFV4(3H)-*RLuc* (infectivity >90%) and measured *RLuc* activity at 7 and 24 hpi as a proxy for viral replication and spread [38]. Results indicated that even as early as 7 hpi inhibition of virus by *Wolbachia* is observed, with a 2–3 fold increase in *RLuc* activity in Jw18Free cells compared to Jw18Wol cells. By 24 hpi this difference is more marked with an 8–12 fold increase in *RLuc* activity in the Jw18Free cells (Fig 2C). Therefore, *Wolbachia* confers antiviral protection against an arbovirus in this system. Furthermore the mechanism by which *Wolbachia* inhibits viral infection must be rapid suggesting either entry of the virus is inhibited or replication/translation are inhibited.

## Suppression of reporter gene expression mediated by activity of the viral subgenomic promoter suggests *Wolbachia* inhibits early replication events

As *Wolbachia* inhibits viral infection or subsequent processes as early as 7 hpi it could be hypothesised that entry of the virus into cells is inhibited, leading to a significant reduction in the number of subsequent replication complexes. In order to test this hypothesis we bypassed viral entry and analysed early translation and replication by transfection of *in vitro* transcribed capped SFV1(3F)*RLuc*-SG-*FFLuc* replicon RNA (Fig 1B). In this SFV-derived replicon RNA, an open reading frame (ORF1) encoding *RLuc* is fused to the non-structural nsP3 and the second, structural ORF(2) has been deleted and replaced with the *FFLuc* ORF (see Materials and Methods). Alphavirus gene expression occurs in separate phases which are linked to replicative processes. Initially ORF1 is translated from the RNA genome giving rise to the nsP proteins, which carry out replicative functions. Then a switch from translation to replication occurs leading to production of a full length antisense copy of the genome, the antigenome. Antisense RNA (which most likely exists in a duplex with the original positive-strand genome) is used as a template for synthesis of new genomes; in addition it carries an internal promoter sequence that directs transcription of a subgenomic mRNA encoding the structural proteins (Fig 1) [39]. Thus, expression of structural proteins (or *FFLuc* marker, Fig 1B) takes place only from these subgenomic RNAs i.e. is fully dependent on the replication process. In contrast, *RLuc* can be produced both by directly translating the replicon that was transfected into the cells as well as by translating new full-length positive strands, generated during RNA replication. Furthermore, in the absence of structural proteins no new virus particles can be formed preventing the spread of infection. It was found that *Wolbachia* results in a significant inhibition of early translation and/or replication independent of normal viral entry, with both *RLuc* and *FFLuc* readouts being significantly lower in Jw18Wol cells compared to Jw18Free cells (Fig 3A and 3B). We cannot rule out that *Wolbachia* may also have an effect on entry which we do not observe in these assays.

As this SFV-derived replicon allows for the separate analysis of transcription and translation from both the genomic and subgenomic promoters and corresponding mRNAs, it allows us to further pinpoint the stage in the replication cycle that is affected by *Wolbachia*. In the *Wolbachia* infected cells we observed a 200–600 fold decrease in *FFLuc* readout, a marker expressed from the RNA produced from subgenomic promoter, which is significantly greater (T-Test $P < 0.0001$) than the ~ 8 fold decrease in *RLuc* readout, a marker produced both from transfected RNAs and full-length positive-strand transcripts from the genomic promoter. This would suggest a clear and early inhibition of establishment of RNA replication. Alternatively, it

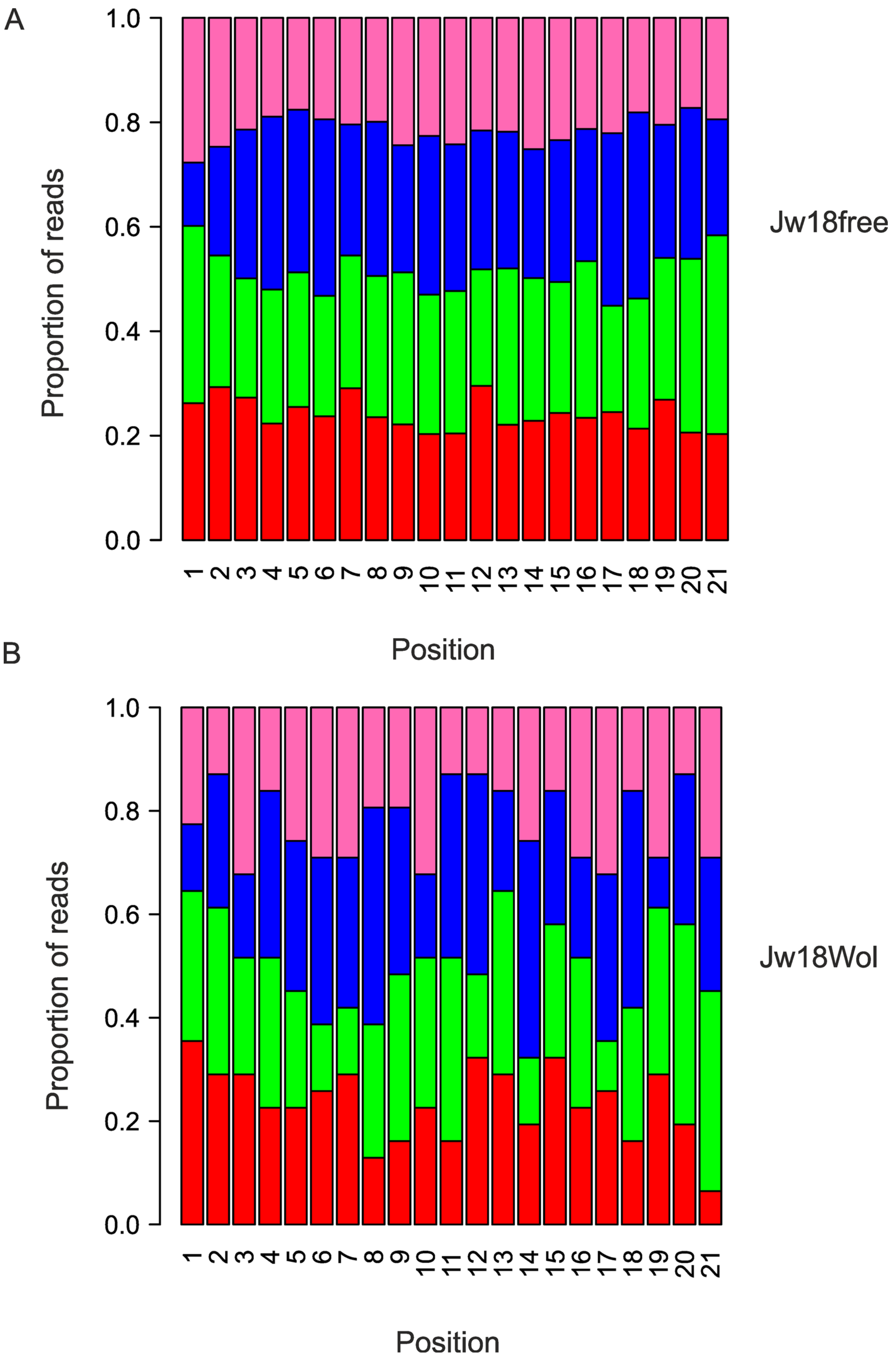
A
1.0
0.8
0.6
0.4
0.2
0.0
Proportion of reads
1 2 3 4 5 6 7 8 9 10 11 12 13 14 15 16 17 18 19 20 21
Position
Jw18free
B
1.0
0.8
0.6
0.4
0.2
0.0
Proportion of reads
1 2 3 4 5 6 7 8 9 10 11 12 13 14 15 16 17 18 19 20 21
Position
Jw18Wol

**Fig 2. Analysis of cell growth and infection dynamics of Jw18Wol and Jw18Free cells infected with SFV.** (A) Jw18Free cells were infected with SFV (3H)-*RLuc* at an MOI of 20. Cells were lysed 4, 8, 12 and 24 hpi and *RLuc* activity determined. The figure represents data from three independent experiments, where each treatment was carried out in triplicate. (B) The density of Jw18Wol and Jw18Free cells 7 and 24 hpi with SFV(3H)-*RLuc* at an MOI of 20; 0 (seed) is 24 h prior to infection. Data represents three independent experiments carried out in duplicate. (C) Jw18Wol and Jw18Free cells were infected at an MOI of 20 with SFV4(3H)-*RLuc* and *RLuc* activity was measured at 7 and 24 hpi. The graph indicates the mean ratio of *RLuc* activity in Jw18Wol and Jw18Free cells, where Jw18Wol at 7hpi and 24 hpi is equal to one. The data represents five independent experiments carried out in duplicate. Error bars represent the standard error of mean in all figures. Stars indicate significance P = <0.05 in T-Test analysis.

doi:10.1371/journal.ppat.1005536.g002

could indicate a specific defect in the production of the subgenomic mRNA. This could occur either by *Wolbachia* directly interfering with replication, or by preventing the translation of proteins required for replication to occur and/or inhibiting the switch from replicase protein translation to RNA replication. Overall the results indicate that early viral RNA translation and/or replication were likely to be inhibited by *Wolbachia*.

To further investigate the effect of *Wolbachia* on viral translation and/or replication we uncoupled viral replicase proteins from viral RNA by the introduction of two plasmids into cells in a SFV transreplicase assay. In this system, one plasmid encodes the viral replicase proteins and the second encodes an RNA template containing the untranslated regions of SFV with either *FFLuc* downstream of the genomic/actin promoter or *Gluc* downstream of the subgenomic promoter. In both cases the expressed sequences are under the control of a *Drosophila* Actin promoter. Upon expression the replicase proteins will bind the RNA template and replication of the reporter construct will take place. Expression of *FFLuc* is under both the control of the Actin promoter and the genomic promoter, therefore due to the high expression from the Actin promoter it is difficult to determine active replication from the genomic reporter. However active replication can be measured from the production of the *Gluc* reporter which is solely under the control of the subgenomic promoter. This system therefore allowed us to determine if production of replicase proteins from mRNA transcribed in the nucleus could overcome *Wolbachia*-mediated protection and determine if the origin of viral RNA is also important. In order to rule out arbitrary effects we also generated a non-replicating replicase with the introduction of a GDD-GAA mutation in the nsP4 protein, thus producing an inactive replicase. Results are shown in Fig 3C and 3D. Activity from the Actin and SFV genomic promoters appeared to be unaffected by the presence of *Wolbachia*, however it should be noted that there is no significant difference between the wildtype and mutant (GAA) replicase or cells where no replicase-expressing plasmid had been transfected. This confirms that the activity we saw here was most likely due to transcription from the Actin promoter and we are unlikely to detect expression from the genomic promoter as the system is at an optimum (Fig 3C). This has also been observed in mammalian systems [40]. As shown in Fig 3D, we observed that *Wolbachia* significantly lowered activity from the subgenomic promoter as *Gluc* activity is lower in Jw18Wol cells compared to Jw18Free. This is in keeping with our previous observation that *Wolbachia* is able to inhibit viral translation and/or replication. Once viral RNA template is produced from the nucleus and transported to the cytoplasm, replication complexes are thought to form as normal. Thus *Wolbachia* was still able to confer protection even when viral replicase/RNA delivery routes were changed. Taken together this data strongly indicated that *Wolbachia* inhibits viral translation and/or replication.

## Virus-derived siRNA profiles support an inhibitory effect of *Wolbachia* on viral replicative processes

A major immune pathway in insects to fight viral infections is the exogenous siRNA pathway which involves the production of virus-derived small interfering RNAs (viRNAs) by the enzyme Dicer-2 acting on virus-derived dsRNA (such as viral replication intermediates) as a

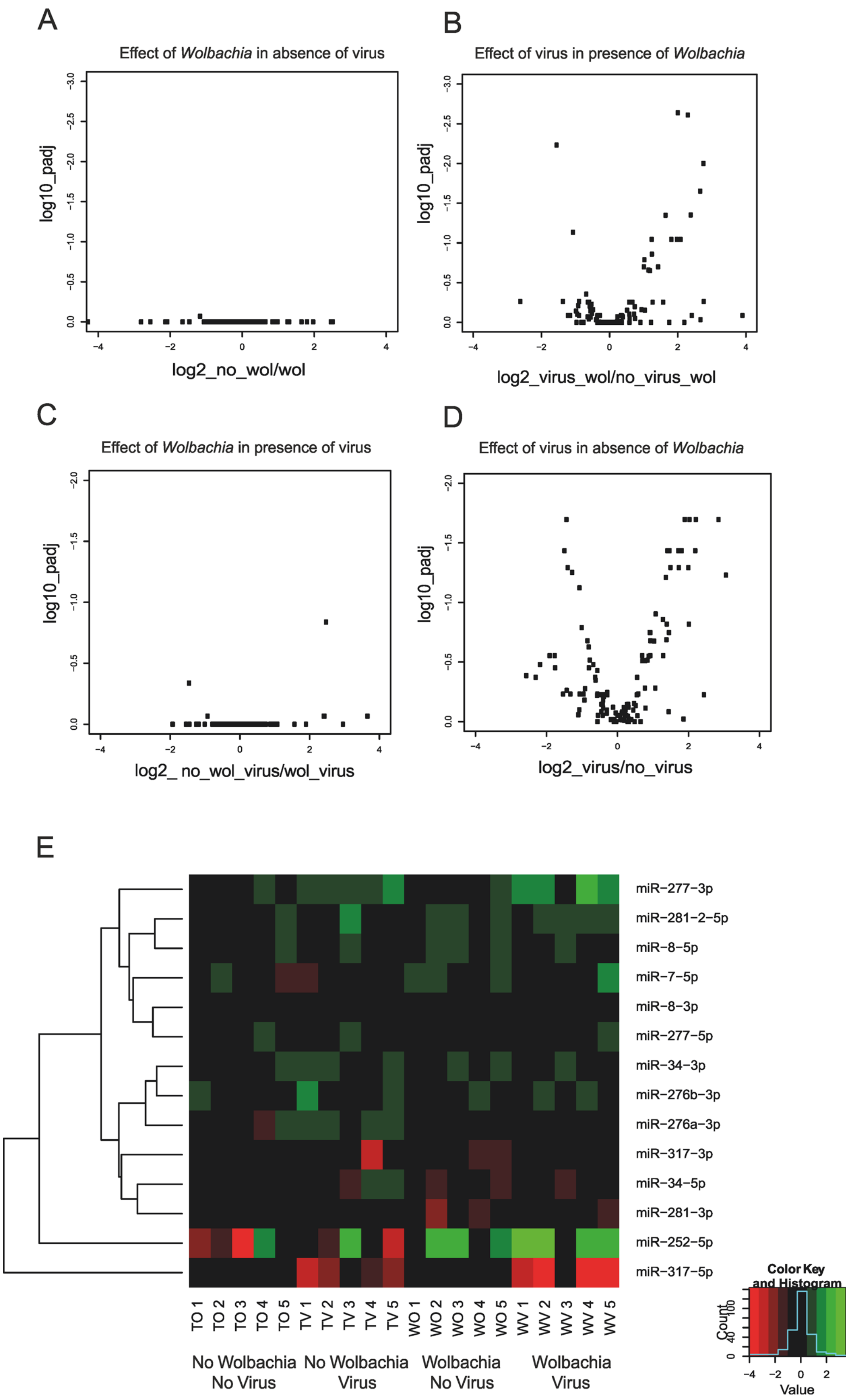
A
Effect of Wolbachia in absence of virus
log10_padj
log2_no_wol/wol
B
Effect of virus in presence of Wolbachia
log10_padj
log2_virus_wol/no_virus_wol
C
Effect of Wolbachia in presence of virus
log10_padj
log2_ no_wol_virus/wol_virus
D
Effect of virus in absence of Wolbachia
log10_padj
log2_virus/no_virus
E
miR−277−3p
miR−281−2−5p
miR−8−5p
miR−7−5p
miR−8−3p
miR−277−5p
miR−34−3p
miR−276b−3p
miR−276a−3p
miR−317−3p
miR−34−5p
miR−281−3p
miR−252−5p
miR−317−5p
TO 1
TO 2
TO 3
TO 4
TO 5
TV 1
TV 2
TV 3
TV 4
TV 5
WO 1
WO 2
WO 3
WO 4
WO 5
WV 1
WV 2
WV 3
WV 4
WV 5
No Wolbachia No Virus
No Wolbachia Virus
Wolbachia No Virus
Wolbachia Virus
Color Key and Histogram
Count
Value

**Fig 3. The effect of *Wolbachia* on SFV replicon and transreplicase activity.** (A, B) Jw18Wol and Jw18Free cells were transfected with *in vitro* transcribed SFV1(3F)*RLuc*-SG-*FFLuc* RNA, and both *RLuc* (A) and *FFLuc* (B) activity was measured 24 h post transfection (hpt). Graphs indicate mean fold change of measurements of luciferase activity where activity in Jw18Wol cells is taken as 1 and represent three independent experiments carried out in triplicate. *RLuc* activity represents translation of genome RNAs whereas *FFLuc* indicates translation of the subgenomic mRNA. Error bars represent standard error of mean. Stars indicate significance where P = <0.05 in T-Test analysis. (C, D) Jw18Wol and Jw18Free cells were transfected with two plasmids: one expressing SFV replicase proteins (wt or mutant, under the control of the *D*. *melanogaster* Actin promoter) and one expressing viral template and both *FFluc* (C) and *Gluc* (D) activity were measured 24 hpt. *FFLuc* activity represents translation of RNA produced from the Actin promoter (and to some extent the genomic promoter) and *Gluc* activity represents translation of RNA produced from the subgenomic promoter following replication. Graphs represent relative luciferase activity and represent three independent experiments carried out in duplicate. Stars indicate significance where P = <0.01 in T-Test analysis.

doi:10.1371/journal.ppat.1005536.g003

substrate [26, 27, 41]. The hallmark of this pathway in insects is the production of 21 nucleotide (nt) viRNAs, a process that has been described in detail for alphavirus infection of mosquitoes and mosquito cells [36, 37, 42]. To test whether the levels of viRNAs were affected by *Wolbachia* we used high-throughput sequencing of 18-33nt small RNAs from Jw18 cells 24hpi with SFV4(3H)-*RLuc*. In Jw18Free cells, small RNAs that were 21 nt long and map to the SFV genome were strongly induced upon viral infection (Wilcoxon unpaired test: $P = 0.008$, Fig 4A, S1A Fig for uninfected controls). 21 nt RNAs mapped equally in both sense and antisense orientations to the viral genome (Fig 4A; $P > 0.1$ Chi-squared test against a uniform distribution). The length and lack of strand bias or first nucleotide bias (Fig 4A) suggest that these small RNAs are generated by the activity of Dicer-2 on virus-derived dsRNA, probably replication intermediates; moreover 21 nt viRNAs were distributed across the viral genome as previously reported for SFV (Fig 5A) [37, 43] and also other arboviruses [26, 27]. Indeed there was no nucleotide bias seen at any position either in the Jw18Free or Jw18Wol cells (S2A and S2B Fig).

If *Wolbachia* infection were to reduce viral infection by upregulating antiviral RNAi we would expect increased viRNA production in the presence of *Wolbachia*. However, whilst 21 nt viRNAs were still present above the background seen in virus-free controls ($P = 0.008$, Wilcoxon unpaired test; Fig 4B and S1B Fig), the amount of viRNAs was strongly reduced on both sense and antisense orientations relative to *Wolbachia*-free cells ($P = 0.008$, Wilcoxon unpaired test; Fig 4A versus 4B). Due to a significant reduction in viral replication, the distribution of 21 nt viRNAs from Jw18Wol cells revealed very few areas of viRNA production (Fig 5B and 5C). RNAs smaller than 21 nt were similar between Jw18Wol and Jw18Free cells in infected cells (Fig 4A and 4B), however it is likely that these smaller species of RNAs are background against the *D*. *melanogaster* genome or degradation products.

This confirmed that *Wolbachia* does not protect against infection by enhancing the production of small RNAs against viruses. Instead, these results are consistent with a model whereby *Wolbachia* interferes with viral replication, leading to a decrease in the levels of viral replication intermediates and therefore a reduction in dsRNA, the substrate available for Dicer-2 and exogenous siRNA pathway induction. This is not surprising as previous studies have shown that *Wolbachia* can still confer antiviral activity in flies mutant for key components of the siRNA pathway [29].

## *Wolbachia* does not alter the expression of miRNAs

Previous studies suggested that *Wolbachia* affects the sensitivity of mosquito cells to viral infection by altering host miRNAs levels [31, 32]. Therefore, we tested whether *Wolbachia* alters the expression of known miRNAs in Jw18 cells. In the absence of virus, no miRNAs had significantly different expression levels in Jw18Wol and Jw18Free cells (Fig 6 and S3A Fig). It is likely

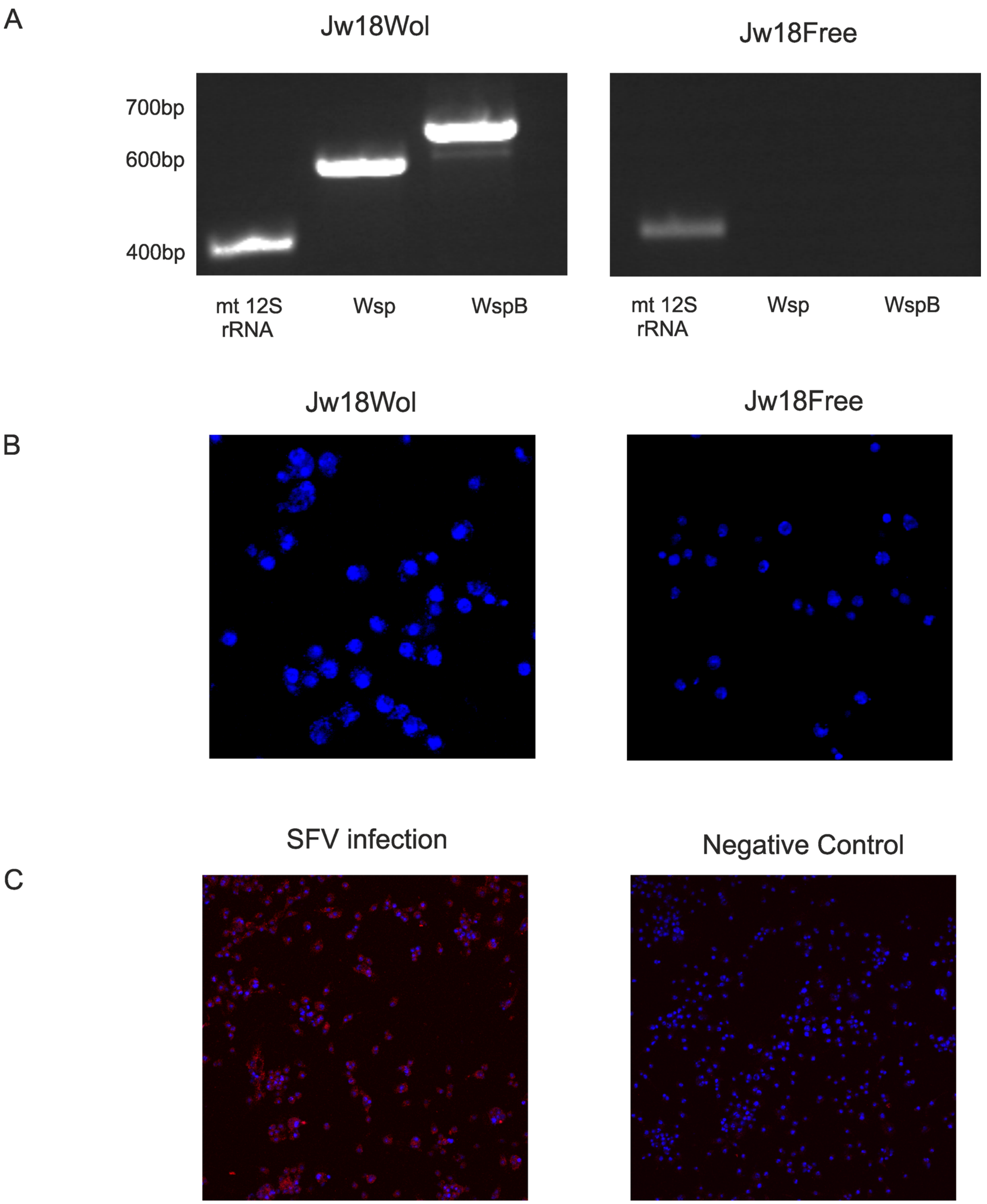


**Fig 4. The effect of *Wolbachia* on the production of virus-derived small interfering RNAs in Jw18 cells.** (A-B) The length and first nucleotide distribution of small RNAs mapping to genome (upper bars, 5′-3′ orientation) or antigenome (lower bars, 3'-5' orientation) of the SFV genome in the (A) absence (Jw18Free) or (B) (Jw18Wol) presence of *Wolbachia*. A = red, C = green, G = blue and T = pink. Data are from 24 hpi with SFV4(3H)-*RLuc*, of Jw18 *D*. *melanogaster* cells. Concatenated data from 5 independent infections are shown in all panels.

doi:10.1371/journal.ppat.1005536.g004

therefore that any differences are not important to *Wolbachia* mediated protection as in our system there are no significantly altered miRNAs between non-infected Jw18Free and Jw18Wol cells.

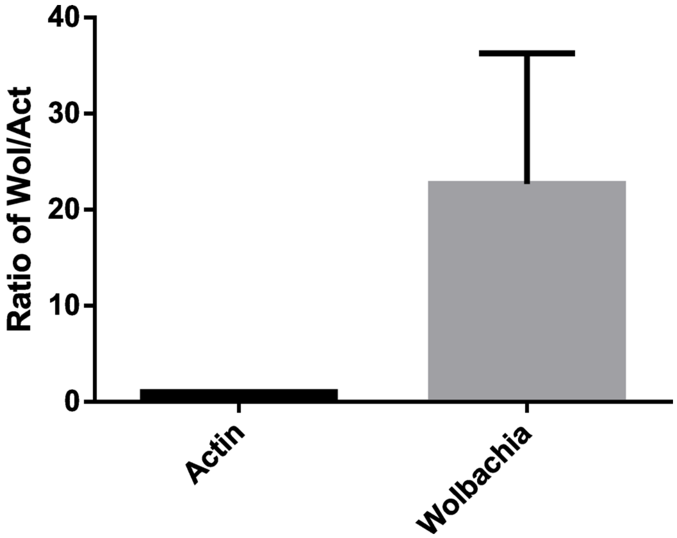


**Fig 5. The effect of *Wolbachia* on the distrubution of virus-derived 21nt interfering RNAs in Jw18 cells.** Distribution of 21 nt long viRNAs along sense (upper bars, black; 5'-3' orientation) or antisense (lower bars, red; 3′-5′ orientation) strands of the SFV genome in the absence (A) (Jw18Free) or presence (B) (Jw18Wol) of *Wolbachia*. A zoomed in version of (B) is shown in (C) for ease of comparison. Data are from 24 hpi with SFV4(3H)-*RLuc*, of Jw18 *D. melanogaster* cells. Concatenated data from 5 independent infections are shown in all panels.

doi:10.1371/journal.ppat.1005536.g005

We next examined whether *Wolbachia* alters the miRNA response to viral infection. We identified a number of miRNAs that significantly changed in abundance when Jw18Free cells were infected with SFV (Figs 6 and S3D; S1 Table). Very similar changes in miRNA expression were seen when cells with *Wolbachia* were infected with virus (Fig 6 and S3B Fig, S1 Table). However, when cells were infected with SFV there were no miRNAs whose abundance was significantly changed by the presence of *Wolbachia* (Fig 6 and S3C Fig). Therefore in *D. melanogaster* cells there is no evidence that *Wolbachia* modulates the constitutive expression of miRNAs or the miRNA-mediated response to infection. The similar miRNA response to virus in cells with and without *Wolbachia* is intriguing as *Wolbachia*-infected cells have greatly reduced levels of virus, and suggests it may reflect a sensitive response to initial infection by the virus.

## Absence of a transcriptional response of *Wolbachia* to viral infection

To investigate whether *Wolbachia* itself might mount an active antiviral response after infection, we tested if there is a transcriptional response of *Wolbachia* to viral infection. We sequenced total RNA from Jw18Wol cells 7 and 24 hpi with SFV4(3H)-*RLuc* virus together with uninfected controls. Over 229 million reads could be mapped to the *D. melanogaster*, *Wolbachia* or SFV transcriptomes (excluding *D. melanogaster* rRNA). Of these 85.6% mapped to *D. melanogaster* and 12.4% mapped to *Wolbachia*. In the virus-infected cells, 3.8% of reads mapped to the SFV genome, and this dropped to 0.03% for cells that were not challenged with the virus. No *Wolbachia* genes were differentially expressed in response to viral infection at either time point (Fig 7B and 7C). There are three reasons to believe that this is a true lack of a transcriptional response and not simply a lack of statistical power. First, across many transcripts we were able to detect a transcriptional response of the cells to SFV in the same experiment (Fig 7D and 7E), and the coverage of many of these differentially expressed transcripts was lower than for many *Wolbachia* transcripts (Fig 7B–7E). Second, we had a very large

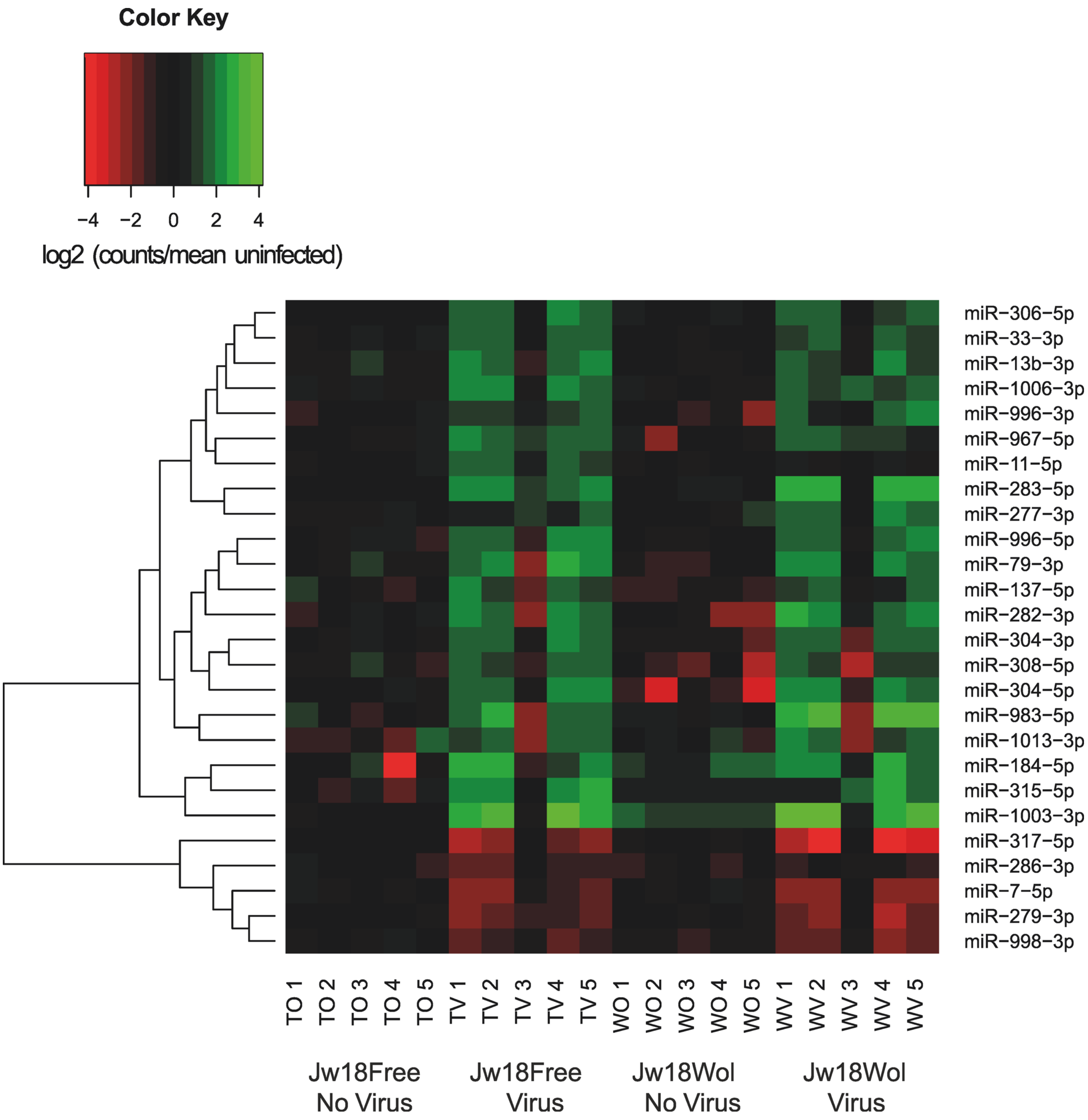


**Fig 6. The effect of *Wolbachia* on *D. melanogaster* miRNA expression in the presence or absence of virus.** Heatmap showing the effects of *Wolbachia* and SFV4(3H)-*RLuc* virus on miRNA abundance. The mean of the No Virus-No *Wolbachia* treatment is set to zero. Only miRNAs that are significantly (Adjusted p value<0.1, Negative Binomial Test) differentially expressed in at least one treatment are shown. Jw18Wol replicate WV 3 was included in analysis; although it appeared to be an outlier as its inclusion did not alter the significance of the data. T = Jw18Free, W = Jw18Wol, V = SFV infection, O = no SFV infection and numbers represent replicate.

doi:10.1371/journal.ppat.1005536.g006

dataset. In each of the 4 treatments about two thirds of the *Wolbachia* transcripts had over 100 reads mapped to them (Fig 7A), which is expected to give good power to detect differential

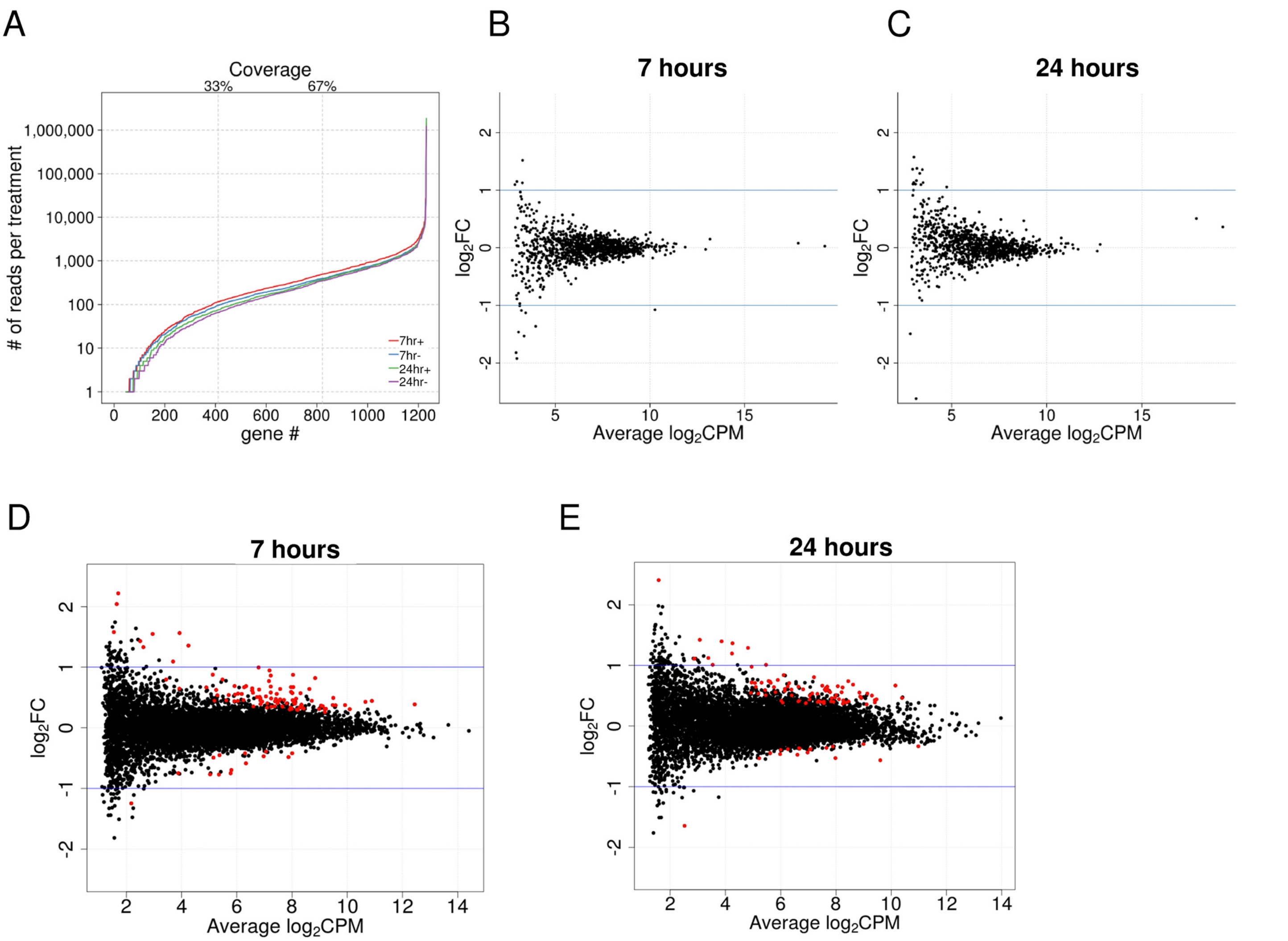


**Fig 7. Analysis of changes in *Wolbachia* and *D. melanogaster* gene expression upon viral infection.** Jw18Wol cells infected with SFV4(3H)-*RLuc* were analyzed. (A) Sequence coverage of *Wolbachia* genes. Genes are arranged from lowest to highest coverage on the X axis. In the legend + refers to the presence of SFV. (B-C) The response to infection ($log_2$ fold expression change) for each *Wolbachia* gene is shown against expression level (average $log_2$ count per million [CPM]) at (B) 7 h and (C) 24 h after infection. (D-E) The response to infection of *D. melanogaster* genes. Genes that significantly change in expression at 10% false discovery rate (FDR) are shown in red (there were no significant induction/repression for *Wolbachia* genes).

doi:10.1371/journal.ppat.1005536.g007

expression. Compared to most published RNAseq experiments ours was a highly replicated experiment involving 40 libraries (biological replicates) across 4 lanes of Illumina HiSeq. Third, if genes with very low-expression are ignored, our estimates of *Wolbachia* gene expression levels in cells with and without SFV were nearly identical (Fig 7B and 7C, $log_2FC \approx 0$). Therefore, the lack of differential expression cannot be attributed to a lack of statistical power.

## Discussion

The bacterial symbiont *Wolbachia* offers an exciting opportunity in the fight against arbovirus transmission by mosquitoes. Several studies have found that it has antiviral activity in key arbovirus mosquito vectors [8, 28, 44, 45]. However, the exact mechanisms behind this activity are poorly understood. In order for *Wolbachia* to be used as a long term and sustainable system to control arbovirus spread, it is critical that we understand these mechanisms. By combining two

powerful model systems–the model arbovirus SFV, and a *Wolbachia*-infected *D. melanogaster* cell line–we were able to show that *Wolbachia* may protect against virus at a very early stage of infection and appeared to block replication and/or translation of viral RNA. This did not involve an active transcriptional response from either the host, the small RNA pathways or *Wolbachia* itself.

Little is known about how *Wolbachia* affects viral replication and dynamics, with studies measuring changes in either the survival of infected insects or viral copy number [5, 6, 17–19]. To investigate the phenomenon in more depth we used the alphavirus SFV, for which virus-encoded reporter genes are known to correspond well to viral replication [38]. We found that *Wolbachia* is able to inhibit viral replication as early as 7 hpi. To our knowledge this is the first indication that *Wolbachia* inhibits viral replication at such an early stage (for example by inhibition of initial translation of incoming RNA or other early replicative processes), as previous studies have focused mainly on days post-infection [5, 17, 19, 46]. This suggests that the mechanism by which *Wolbachia* is conferring antiviral activity is either fast acting or is already present upon viral infection i.e. intrinsic. This is important as for *Wolbachia* to be used successfully as a control mechanism for arbovirus transmission, control of viral replicative processes at an early stage could be vital, as it would not allow the virus to replicate to high levels allowing for dissemination.

The SFV life cycle can be divided into entry, initial translation of incoming viral genomes, switch from translation to replication, RNA replication, translation of structural proteins from subgenomic RNA, assembly and exit from cells. Understanding where in this lifecycle *Wolbachia* acts is vital to understanding the mechanisms behind its antiviral activity. This can often prove difficult to investigate as little is understood about SFV (and arbovirus) replication in insect cells compared to vertebrate cells. By utilising SFV reporter viruses and replicon constructs we can begin to deconstruct the replication cycle. The very early stage at which SFV was inhibited and the fact that bypassing viral entry still resulted in viral inhibition by *Wolbachia* suggests that *Wolbachia* is likely blocking an early post-entry stage in the replication cycle. We can further dissect how *Wolbachia* affects the replication cycle due to the presence of a sub-genomic promoter in alphaviruses [39, 47]. Expression from the first ORF of SFV does not require viral replication (though due to an increase of genomic RNA copy number, it is dramatically increased when replication occurs), as positive-sense RNA virus genomes can act as mRNA immediately after infection. In contrast translation and protein expression from the second ORF of SFV requires a full round of replication in order for the sub-genomic promoter to become available. Therefore by utilising the replicon SFV expressing *RLuc* reporter directly from genomic RNA and *FFLuc* via activity of the subgenomic promoter we could test whether *Wolbachia* is affecting the replication of viral RNA. Our results showed that viral replication and/or translation of replicon were inhibited by *Wolbachia*. This could occur either by *Wolbachia* directly interfering with replication, or by inhibiting the translation of viral proteins required for this to occur and/or inhibiting the switch from translation to replication. Further to this we showed by using a SFV transreplicase system and uncoupling the replicase proteins from template RNA that there was significant inhibition of the subgenomic promoter and thus viral translation and/or replication. Thus providing replicase proteins either directly or separately did not overcome the inhibition phenotype. These results indicate that the origin of viral RNA (transcription in the nucleus in the transreplicase system) was also not important to *Wolbachia*-mediated antiviral activity.

This block in translation/replication in the presence of the endosymbiont was backed by analysis of the induction of small RNA responses, which reveal that *Wolbachia*-infected cells did not show higher production of viRNAs which are derived from the dsRNA generated during viral replication (see below).

One possible way in which *Wolbachia* could protect against viruses would be by the bacterium mounting an active antiviral response following infection. If this were the case, it would likely be reflected in a transcriptional response of the endosymbiont to the virus. However, in a very large dataset we did not find a single *Wolbachia* gene that was differentially regulated in response to viral infection. This is consistent with the very early inhibition of viral replication, which suggests that the antiviral mechanism is constitutively present before the virus infects the cell and is thus intrinsic.

Previous studies have indicated that a host miRNA response may be responsible for *Wolbachia*'s antiviral activity [30, 31, 48], but we found no support for this hypothesis in our system. In virus-free mosquitoes *Wolbachia* changes the expression of a number of miRNAs, but we found no such differences in our cell line (S3E Fig). Previous studies did not analyze concurrent infections of both *Wolbachia* and virus [30, 31, 48]. When we did this we found a marked miRNA response to the virus, but this was unaltered by the presence of *Wolbachia*. It is surprising that we still see host miRNA response to viral infection in the presence of *Wolbachia*, as the level of SFV infection is extremely low. This suggests that miRNA response may be due to early events in viral replication such as virion binding and/or entry or that this response requires very little viral protein synthesis/RNA replication to be initiated. Previous studies have indicated that the viRNA pathway is not required for *Wolbachia*'s antiviral activity [29]. Our data supports this, with a strong reduction in 21 nt viRNAs mapping to the SFV genome and antigenome when *Wolbachia* is present. This suggests that viral replication is inhibited so significantly that very few viRNAs are produced, rather than *Wolbachia* inducing an antiviral RNAi response. Further studies utilising this system would be beneficial to the field. For example little is known about the possibility of viruses mutating to overcome *Wolbachia* mediated protection as long term studies are lacking. A cell-based assay offers an ideal opportunity to look at virus evolution over the long term in such associations. In addition to this it would be interesting to look at the effect on other viruses in our system as other studies have indicated that even within the same family *Wolbachia* can have different effects on viruses [49]. In *Drosophila* studies *Wolbachia* is also known to protect against FHV without lowering viral titres. It would be interesting to look at this in the context of our findings, as it may suggest another mechanism by which *Wolbachia* can confer antiviral activity [6].

In conclusion we have developed a powerful new system to study the replication dynamics of SFV in *Wolbachia*-infected *D. melanogaster* cells. While the exact mechanism of the antiviral response remains unknown, current data is consistent with a 'passive' mechanism such as competition for resources or space, or by *Wolbachia* remodelling the intracellular environment. While effects of *Wolbachia* on entry or exit cannot be excluded, our data point to an effect on translation and/or replication at least for this model alphavirus. Considering the broad antiviral effects of *Wolbachia* across *Drosophila* and mosquito species, it is tempting to propose a model of inhibition that relies on similar intrinsic mechanisms rather than diverse processes such as miRNA regulation or immune responses. The data presented in this study point towards such an antiviral mode of action by *Wolbachia* endosymbionts.

## Materials and Methods

### Cell culture

The *Wolbachia*-infected *D. melanogaster* cell line Jw18Wol (obtained from W. Sullivan, L. Serbus, A. Debec) has been described elsewhere [34]. A corresponding tetracycline cured line (Jw18Free) was created by treating cells with 10 μg/ml of tetracycline for 4 passages, cells were then tested for *Wolbachia* by PCR and DAPI staining and if the infection was cleared cells were left for 5 more passages in order to eliminate tetracycline effects. Cells were maintained at 24°C

in Shields and Sanger media (Sigma) supplemented with 10% fetal calf serum (FCS) and 10% Penicillin/Streptomycin (Pen/Strep). Cells were checked every four passages for the presence of *Wolbachia* by PCR using two separate primer pairs as described previously [6]; cells were stained with DAPI in order to visualise *Wolbachia* content (shown as dots in cytoplasm), density in Jw18Wol cells was consistent with previous observations with ~90% of cells infected (S4 Fig). qPCR was also carried out in order to determine *Wolbachia* density. Standard curve analysis was carried out and normalised to an Actin endogenous control. Primers used were as follows, Actin5CF_ GACGAAGAAGTTGCTGCTCTGGTTG Actin5CR TGAGGATACCA CGCTTGCTCTGC and WolF GTTTGCAATACAACGGTGAA WolR CAACCCTGA TGTCGTCCATT. qPCR was carried out using the ABI Fast SYBR Green Master Mix, as per manufactures guidelines, on an ABI 750 Fast machine. Results indicated that when compared to Actin endogenous control there is ~22 times more *Wolbachia* (S5 Fig), suggesting a density of at least 100% with more than one copy number per cell as is seen in the DAPi staining.

## Virus production and *in vitro* transcription of replicon RNA

SFV4(3H)-*RLuc* virus was grown and cultivated as described previously [37]. For replicon production, pSFV1(3F)*RLuc*-SG-*FFLuc* plasmid (details available on request) was linearized with SpeI and purified using the PCR product purification kit (Roche). 1 μg of linearized DNA was *in vitro* transcribed using MEGAscript SP6 polymerase kit (Ambion) in the presence of cap analog (Ambion).

## SFV transreplicase system: construction of replicase and template expression constructs

pAc51-V5-His backbone was used to construct the plasmid expressing the replicase of SFV. First, the multiple coning site of the plasmid was replaced by a polylinker sequence TTCGAA TATGGATCCTATTTAATTAATATCCTAGG (recognition sites of Bsp119I and PacI are underlined). Second, Bsp119I and PacI adapters were added to the 5' and 3' ends of the sequence encoding SFV replicase, respectively, by using PCR and subcloning procedures. Finally, the sequence encoding SFV replicase was inserted into modified pAc51 vector using Bsp119I and PacI restriction sites. The resulting plasmid was designated as pAct51-SFVRepl. In order to obtain plasmid encoding for a polymerase defective form of SFV replicase, the conserved GDD motif in nsP4 was altered to GAA using PCR-based mutagenesis and subcloning procedures; the resulting plasmid was designated pAc51-SFVRepl-GAA.

In order to obtain a plasmid for expression of template RNA for the SFV replicase the polylinker and terminator regions of pAc51-V5-His were replaced with a synthetic DNA fragment consisting from the first 280 nucleotides of SFV genome (including EcoRV restriction site, nucleotides 275–280) followed by the sequence TATGGATCCTATGGCGCGCCGTCGAC (recognition sites BamHI, BssH2 and SalI underlined). The replacement was performed in such a way that the 5' end of the SFV genome was positioned exactly downstream of last start site of actin promoter. The following sequences were added using synthetic DNA fragments (GenScript, USA) and subcloning procedures: a) sequence encoding for firefly luciferase (*FFLuc*), placed in frame with N-terminal fragment of SFV nsP1 (amino acid residues 1–65, encoded by the 5' region of SFV genome); b) SFV subgenomic promoter spanning from position -145 to +51 with respect of the start site of SFV subgenomic RNA; c) sequence encoding for *Gaussia* luciferase (*Gluc*); d) 3' UTR of SFV followed by 69 adenine residues; e) negative strand ribozyme of hepatitis delta virus; e) late termination region of simian virus 40. Elements a, b, c and d were separated from each other by recognition sequences of EcoRV, ApaI and Bsp119I nucleases, respectively. The plasmid was designated as pAc51-Temp-Fluc2-SG-Gluc.

## Infection of cells with SFV

Cells were plated out at a density of $3.5x10^5$ cells/well 24 h prior to infection in a 24 well plate. For infection, virus was diluted in Shields and Sanger media (Sigma) with no FCS. Virus was titred as described [37] and an MOI of 20 was shown to give an infectivity of over 90% in Jw18Free cells (S4C Fig). Cells were incubated for 3 h before media was replaced with fresh media supplemented with 10% FCS and Pen/Strep. Samples were collected at time points as indicated. Virus free cells were mock infected by treating the same as infected cells but without the addition of virus to the media.

## Immunofluorescence detection of *Wolbachia* and SFV

*Wolbachia* was detected using the nuclear stain DAPI. Briefly cells were fixed in 4% paraformaldehyde, permeabilized and covered in Vectashield containing DAPI. *Wolbachia* was indicated by the presence staining outside of the nucleus which was absent in tetracycline treated cells. SFV was detected as described previously using an antibody against the replicative protein nsP2 [50].

## Transfection of cells with SFV1(3F)*RLuc*-SG-*FFLuc* replicon RNA or SFV transreplicase plasmids

Cells were plated out at a density of $3.5x10^5$ cells/well 24 h prior to transfection in a 24 well plate. Cells were transfected with 1 μl of *in vitro* transcribed RNA using Fugene in Shields and Sanger media minus FCS. Cells were incubated for 2 h before media was replaced with fresh media supplemented with 10% FCS and Pen/Strep. For transreplicase experiments 300 ng of each plasmid was transfected into cells as described above.

## Luciferase assays

Cells were lysed in passive lysis buffer and luciferase readings carried out using the Dual Luciferase Kit (Promega). Luciferase activities were determined on a Glomax Multi+ Microplate Multimode reader (Promega).

## Sequencing and analysis of small RNAs

Cells were infected with SFV as described above. At 7 and 24 hpi cells were lysed in 1 ml of Trizol solution. Small RNA libraries were prepared according to the Illumina method using the Truseq small RNA Preparation kit. We made a small adjustment to the manufacturer's protocol to include a ribosomal RNA blocking step prior to ligation of 5′ adapter and reverse transcription in order to eliminate the abundant 30 nt *D. melanogaster* 2S rRNA, as specified in [51]. Small RNA libraries were then sequenced on one lane of a HiSeq 2000. High throughput sequencing data was processed and aligned to the viral genome as described previously [52]. miRNAs were mapped to *D. melanogaster* miRNAs downloaded from miRBase [53] using a custom Perl script and analysed to identify statistically significant changes in expression according to the negative binomial distribution using the R package DESeq as described [54]. All data processing was carried out in the R statistical environment. Sequence data has been submitted to the Sequence Read Archive (SRA) under accession number PRJEB9710.

## Sequencing and analysis of total RNA

*Wolbachia*-positive Jw18Wol cells were infected with SFV at an MOI of 20 as described above. 7 and 24 hpi cells were lysed in 1 ml of Trizol; total RNA was extracted using the Direct-Zol MiniPrep kit (Zymo). The extracted RNA was then treated with TURBO DNase (Ambion) and

purified using the RNA Clean and Concentrator kit (Zymo). *D. melanogaster* ribosomal RNA was then depleted using the Ribo-Zero Gold Magnetic kit (Human/Mouse/Rat, Epicentre). Libraries of the rRNA-depleted total RNA were prepared at The Genome Analysis Center (Norwich) with the Truseq RNA Sample Preparation kit (Illumina) and sequenced in 2 lanes on a HiSeq 2000. Sequence data has been submitted to the Sequence Read Archive (SRA) under accession number PRJEB10681. Sequences were quality trimmed from the 5′ and 3′ ends using Trimmomatic version 0.30 [55] when average quality scores in sliding windows of 4 base pairs dropped below 20 or when the quality score at the beginning or end of the read dropped below 20. Sequences less than 25 bp in length were discarded. Reads were aligned to the transcriptomes of *Wolbachia* strain *w*Mel (Genbank accession number: GCA_000008025.1) and *D. melanogaster* (BDGP v. 5.25), and to the genome of SFV (Genbank: KP699763.1). Alignments were performed using Bowtie2 version 2.1.0 [56] with default parameters, and splicing was allowed in *D. melanogaster* using TopHat2 version 2.0.9 [57] with default parameters and no novel junctions allowed. The numbers of reads per transcript were counted using HTSeq [58] for *Wolbachia* and *D. melanogaster*. Differential expression analysis was performed using edgeR [59]. Lowly expressed genes were filtered out by requiring that each gene have at least 1 count per million in at least 8 samples. Differential expression in response to viral infection was measured separately at 7 and 24 hpi. Significance was assessed using exact tests [59] with a FDR of 10%.

## Supporting Information

**S1 Fig. Analysis of small RNAs profiles in virus-free cells in the different treatments.** This data is the virus-free controls for Fig 4A and 4B in the main text. The length and first nucleotide distribution of small RNAs mapping to SFV genome (upper bars, 5′-3′ orientation) or anti-genome (lower bars, 3'-5' orientation) at 24 h post mock infection of *D. melanogaster* cells in the absence (A) (Jw18Free) or presence (B) (Jw18Wol) of *Wolbachia* are shown. Concatenated data from 5 independent infections are shown. A = red, C = green, G = blue and T = pink.
(TIF)

**S2 Fig. The effect of *Wolbachia* on the distribution of nucleotides in virus-derived small interfering RNAs (viRNAs).** Mapping of nucleotide distribution of small RNAs mapping to the SFV genome in the (A) (Jw18Free) absence or (B) (Jw18Wol) presence of *Wolbachia*.
A = red, C = green, G = blue and T = pink.
(TIF)

**S3 Fig. The expression of miRNAs in *D. melanogaster* Jw18 cells with (Jw18Wol) and without (Jw18Free) *Wolbachia*, and with and without virus (SFV) infection.** Panels (A-D) are volcano plots summarizing the differential expression of miRNAs between pairs of treatments. The Y axis is the $\log_{10}$ of the FDR corrected *P* value. The X axis is the change in expression on a $\log_2$ scale. (E) Heatmap showing the relative expression of miRNAs homologous to those reported to be affected by *Wolbachia* infection of mosquito cells [48]. T = Jw18Free, W = Jw18Wol, V = SFV infection, O = no SFV infection and numbers represent replicate.
(TIF)

**S4 Fig. Detection of *Wolbachia* infection by DAPI staining.** Cells were tetracycline treated or not and stained with DAPI in order to visualize *Wolbachia* infection; tetracycline-treated cells are referred to as Jw18Free. (A) Cells were checked for the presence of *Wolbachia* by PCR using two separate primer pairs as described previously [6]. (B) Cells were then stained with DAPI in order to visualise *Wolbachia* content, density was consistent with previous observations with ~90% of cells infected. (C) Jw18Free cells were infected with an MOI of 20 calculated

in BHK cells and stained with SFV NSP2 antibody [50] in order to determine infection rate. Cells positive for SFV were shown to have an infection rate >90%.
(TIF)

**S5 Fig. Calculation of *Wolbachia* density by qPCR.** *Wolbachia* density was calculated as a ratio to the endogenous control Actin 5C (Wol/Act), where it is assumed there is one copy of actin per cell. Experiments were carried out in triplicate with two biological replicates. Error bar indicates standard deviation.
(TIF)

**S1 Table. List of miRNAs significantly differentially expressed in response to SFV in cells both with and without *Wolbachia*.** Table indicates miRNAs found to be significantly differentially expressed (Adjusted p value<0.1, Negative Binomial Test) upon SFV infection in either cells positive (Jw18Wol) or negative (Jw18Free) for *Wolbachia*. Black means significantly upregulated and red significantly down regulated.
(DOCX)

## Acknowledgments

We thank Sylviane Moss for support with high-throughput sequencing. We thank Isabelle Dietrich for helpful discussion. We also thank William Sullivan, Laura Serbus and Alain Debec for sharing the Jw18 cells.

## Author Contributions

Conceived and designed the experiments: SMR AK FMJ JM PS EAM ES. Performed the experiments: SMR JM MM PJ PS MV. Analyzed the data: SMR JM FMJ PJ PS AK. Contributed reagents/materials/analysis tools: AL AM EAM. Wrote the paper: SMR AK FMJ PS JM.

## References

1. Kean J, Rainey S, McFarlane M, Donald C, Schnettler E, Kohl A, et al. Fighting Arbovirus Transmission: Natural and Engineered Control of Vector Competence in Aedes Mosquitoes. Insects. 2015; 6(1):236. doi: 10.3390/insects6010236 PMID: 26463078
2. Weaver SC, Reisen WK. Present and future arboviral threats. Antiviral Res. 2010; 85(2):328–45. Epub 2009/10/28. doi: S0166-3542(09)00495-1 [pii] doi: 10.1016/j.antiviral.2009.10.008 PMID: 19857523; PubMed Central PMCID: PMC2815176.
3. Rainey SM, Shah PN, Kohl A, Dietrich I. Understanding the Wolbachia-mediated inhibition of arboviruses in mosquitoes: progress and challenges. J Gen Virol. 2013; 16(10):057422–0.
4. Iturbe-Ormaetxe I, Walker T, SL ON. Wolbachia and the biological control of mosquito-borne disease. EMBO Rep. 2011; 12(6):508–18. doi: 10.1038/embor.2011.84 PMID: 21546911; PubMed Central PMCID: PMC3128286.
5. Hedges LM, Brownlie JC, O'Neill SL, Johnson KN. Wolbachia and virus protection in insects. Science. 2008; 322(5902):702. Epub 2008/11/01. doi: 10.1126/science.1162418 PMID: 18974344.
6. Teixeira L, Ferreira A, Ashburner M. The bacterial symbiont Wolbachia induces resistance to RNA viral infections in Drosophila melanogaster. PLoS Biol. 2008; 6(12):e2. PMID: 19222304. doi: 10.1371/journal.pbio.1000002
7. Walker T, Johnson PH, Moreira LA, Iturbe-Ormaetxe I, Frentiu FD, McMeniman CJ, et al. The wMel Wolbachia strain blocks dengue and invades caged Aedes aegypti populations. Nature. 2011; 476(7361):450–3. Epub 2011/08/26. doi: 10.1038/nature10355 PMID: 21866159.
8. Moreira LA, Iturbe-Ormaetxe I, Jeffery JA, Lu G, Pyke AT, Hedges LM, et al. A Wolbachia symbiont in Aedes aegypti limits infection with dengue, Chikungunya, and Plasmodium. Cell. 2009; 139(7):1268–78. Epub 2010/01/13. doi: 10.1016/j.cell.2009.11.042 PMID: 20064373.
9. McMeniman CJ, Lane RV, Cass BN, Fong AW, Sidhu M, Wang YF, et al. Stable introduction of a life-shortening Wolbachia infection into the mosquito Aedes aegypti. Science. 2009; 323(5910):141–4. Epub 2009/01/03. doi: 323/5910/141 [pii] doi: 10.1126/science.1165326 PMID: 19119237.

10. Sinkins SP. Wolbachia and cytoplasmic incompatibility in mosquitoes. Insect Biochem Mol Biol. 2004; 34(7):723–9. Epub 2004/07/10. doi: 10.1016/j.ibmb.2004.03.025 PMID: 15242714.
11. Hoffmann AA, Montgomery BL, Popovici J, Iturbe-Ormaetxe I, Johnson PH, Muzzi F, et al. Successful establishment of Wolbachia in Aedes populations to suppress dengue transmission. Nature. 2011; 476 (7361):454–7. Epub 2011/08/26. doi: 10.1038/nature10356 PMID: 21866160.
12. Hoffmann AA, Iturbe-Ormaetxe I, Callahan AG, Phillips BL, Billington K, Axford JK, et al. Stability of the wMel Wolbachia Infection following invasion into Aedes aegypti populations. PLoS Negl Trop Dis. 2014; 8(9):e3115. doi: 10.1371/journal.pntd.0003115 PMID: 25211492; PubMed Central PMCID: PMC4161343.
13. Frentiu FD, Zakir T, Walker T, Popovici J, Pyke AT, van den Hurk A, et al. Limited dengue virus replication in field-collected Aedes aegypti mosquitoes infected with Wolbachia. PLoS Negl Trop Dis. 2014; 8 (2):e2688. doi: 10.1371/journal.pntd.0002688 PMID: 24587459; PubMed Central PMCID: PMC3930499.
14. Osborne SE, Iturbe-Ormaetxe I, Brownlie JC, O'Neill SL, Johnson KN. Antiviral protection and the importance of Wolbachia density and tissue tropism in Drosophila simulans. Applied and environmental microbiology. 2012; 78(19):6922–9. Epub 2012/07/31. doi: 10.1128/AEM.01727-12 PMID: 22843518; PubMed Central PMCID: PMC3457512.
15. Osborne SE, Leong YS, O'Neill SL, Johnson KN. Variation in antiviral protection mediated by different Wolbachia strains in Drosophila simulans. PLoS Pathog. 2009; 5(11):e1000656. Epub 2009/11/17. doi: 10.1371/journal.ppat.1000656 PMID: 19911047; PubMed Central PMCID: PMC2768908.
16. Martinez J, Longdon B, Bauer S, Chan YS, Miller WJ, Bourtzis K, et al. Symbionts commonly provide broad spectrum resistance to viruses in insects: a comparative analysis of Wolbachia strains. PLoS Pathog. 2014; 10(9):e1004369. doi: 10.1371/journal.ppat.1004369 PMID: 25233341; PubMed Central PMCID: PMC4169468.
17. Bian G, Xu Y, Lu P, Xie Y, Xi Z. The endosymbiotic bacterium Wolbachia induces resistance to dengue virus in Aedes aegypti. PLoS Pathog. 2010; 6(4):e1000833. Epub 2010/04/07. doi: 10.1371/journal.ppat.1000833 PMID: 20368968; PubMed Central PMCID: PMC2848556.
18. Lu P, Bian G, Pan X, Xi Z. Wolbachia induces density-dependent inhibition to dengue virus in mosquito cells. PLoS Negl Trop Dis. 2012; 6(7):e1754. Epub 2012/08/01. doi: 10.1371/journal.pntd.0001754 PNTD-D-12-00216 [pii]. PMID: 22848774; PubMed Central PMCID: PMC3404113.
19. Frentiu FD, Robinson J, Young PR, McGraw EA, O'Neill SL. Wolbachia-Mediated Resistance to Dengue Virus Infection and Death at the Cellular Level. PloS one. 2010; 5(10). doi: ARTN e13398 doi: 10.1371/journal.pone.0013398 PMID: ISI:000283043700006.
20. Caragata EP, Rances E, Hedges LM, Gofton AW, Johnson KN, O'Neill SL, et al. Dietary cholesterol modulates pathogen blocking by wolbachia. PLoS Pathog. 2013; 9(6):e1003459. Epub 2013/07/05. doi: 10.1371/journal.ppat.1003459 PPATHOGENS-D-12-02513 [pii]. PMID: 23825950; PubMed Central PMCID: PMC3694857.
21. Rances E, Ye YH, Woolfit M, McGraw EA, O'Neill SL. The relative importance of innate immune priming in Wolbachia-mediated dengue interference. Plos Pathogens. 2012; 8(2):e1002548. Epub 2012/03/03. doi: 10.1371/journal.ppat.1002548 PMID: 22383881; PubMed Central PMCID: PMC3285598.
22. Tchankouo-Nguetcheu S, Khun H, Pincet L, Roux P, Bahut M, Huerre M, et al. Differential protein modulation in midguts of Aedes aegypti infected with chikungunya and dengue 2 viruses. PloS one. 2010; 5 (10). Epub 2010/10/20. doi: 10.1371/journal.pone.0013149 PMID: 20957153; PubMed Central PMCID: PMC2950154.
23. Kambris Z, Blagborough AM, Pinto SB, Blagrove MS, Godfray HC, Sinden RE, et al. Wolbachia stimulates immune gene expression and inhibits plasmodium development in Anopheles gambiae. Plos Pathogens. 2010; 6(10):e1001143. Epub 2010/10/16. doi: 10.1371/journal.ppat.1001143 PMID: 20949079; PubMed Central PMCID: PMC2951381.
24. Rances E, Johnson TK, Popovici J, Iturbe-Ormaetxe I, Zakir T, Warr CG, et al. The Toll and Imd Pathways Are Not Required for Wolbachia-Mediated Dengue Virus Interference. Journal of virology. 2013; 87(21):11945–9. Epub 2013/08/30. doi: 10.1128/JVI.01522-13 PMID: 23986574.
25. Bourtzis K, Pettigrew MM, O'Neill SL. Wolbachia neither induces nor suppresses transcripts encoding antimicrobial peptides. Insect molecular biology. 2000; 9(6):635–9. Epub 2000/12/21. PMID: 11122472.
26. Donald CL, Kohl A, Schnettler E. New insights into control of arbovirus replication and spread by insect RNA interference pathways. Insects. 2012; 3(2):511–31. doi: 10.3390/insects3020511 PMID: 26466541
27. Blair CD, Olson KE. The role of RNA interference (RNAi) in arbovirus-vector interactions. Viruses. 2015; 7(2):820–43. doi: 10.3390/v7020820 PMID: 25690800; PubMed Central PMCID: PMC4353918.

28. Glaser RL, Meola MA. The native Wolbachia endosymbionts of Drosophila melanogaster and Culex quinquefasciatus increase host resistance to West Nile virus infection. PloS one. 2010; 5(8):e11977. Epub 2010/08/12. doi: 10.1371/journal.pone.0011977 PMID: 20700535; PubMed Central PMCID: PMC2916829.

29. Hedges LM, Yamada R, O'Neill SL, Johnson KN. The siRNA pathway is not essential for Wolbachia-mediated antiviral protection in Drosophila. Applied and environmental microbiology. 2012; 78 (18):6773–6. Epub 2012/07/13. doi: 10.1128/aem.01650-12 PMID: 22798369

30. Osei-Amo S, Hussain M, O'Neill SL, Asgari S. Wolbachia-induced aae-miR-12 miRNA negatively regulates the expression of MCT1 and MCM6 genes in Wolbachia-infected mosquito cell line. PloS one. 2012; 7(11):e50049. Epub 2012/11/21. doi: 10.1371/journal.pone.0050049 PMID: 23166816; PubMed Central PMCID: PMC3500346.

31. Hussain M, Frentiu FD, Moreira LA, O'Neill SL, Asgari S. Wolbachia uses host microRNAs to manipulate host gene expression and facilitate colonization of the dengue vector Aedes aegypti. Proc Natl Acad Sci U S A. 2011; 108(22):9250–5. Epub 2011/05/18. doi: 10.1073/pnas.1105469108 [pii]. PMID: 21576469; PubMed Central PMCID: PMC3107320.

32. Zhang G, Hussain M, O'Neill SL, Asgari S. Wolbachia uses a host microRNA to regulate transcripts of a methyltransferase, contributing to dengue virus inhibition in Aedes aegypti. Proc Natl Acad Sci U S A. 2013; 110(25):10276–81. Epub 2013/06/05. doi: 10.1073/pnas.1303603110 PMID: 23733960; PubMed Central PMCID: PMC3690878.

33. Lucas K, Raikhel AS. Insect microRNAs: biogenesis, expression profiling and biological functions. Insect Biochem Mol Biol. 2013; 43(1):24–38. doi: 10.1016/j.ibmb.2012.10.009 PMID: 23165178; PubMed Central PMCID: PMC3534889.

34. Serbus LR, Landmann F, Bray WM, White PM, Ruybal J, Lokey RS, et al. A Cell-Based Screen Reveals that the Albendazole Metabolite, Albendazole Sulfone, Targets Wolbachia. Plos Pathogens. 2012; 8 (9). doi: ARTN e1002922 doi: 10.1371/journal.ppat.1002922 PMID: ISI:000309816500028.

35. Rodriguez-Andres J, Rani S, Varjak M, Chase-Topping ME, Beck MH, Ferguson MC, et al. Phenoloxidase activity acts as a mosquito innate immune response against infection with Semliki Forest virus. PLoS Pathog. 2012; 8(11):e1002977. Epub 2012/11/13. doi: 10.1371/journal.ppat.1002977 PPATHOGENS-D-12-01166 [pii]. PMID: 23144608; PubMed Central PMCID: PMC3493465.

36. Schnettler E, Donald CL, Human S, Watson M, Siu RW, McFarlane M, et al. Knockdown of piRNA pathway proteins results in enhanced Semliki Forest virus production in mosquito cells. J Gen Virol. 2013; 94(Pt 7):1680–9. doi: 10.1099/vir.0.053850–0 PMID: 23559478; PubMed Central PMCID: PMC3709635.

37. Siu RW, Fragkoudis R, Simmonds P, Donald CL, Chase-Topping ME, Barry G, et al. Antiviral RNA interference responses induced by Semliki Forest virus infection of mosquito cells: characterization, origin, and frequency-dependent functions of virus-derived small interfering RNAs. J Virol. 2011; 85 (6):2907–17. doi: 10.1128/JVI.02052-10 PMID: 21191029; PubMed Central PMCID: PMC3067965.

38. Fragkoudis R, Chi Y, Siu RWC, Barry G, Attarzadeh-Yazdi G, Merits A, et al. Semliki Forest virus strongly reduces mosquito host defence signaling. Insect molecular biology. 2008; 17(6):647–56. doi: 10.1111/j.1365-2583.2008.00834.x PMID: ISI:000260729100006.

39. Rupp JC, Sokoloski KJ, Gebhart NN, Hardy RW. Alphavirus RNA synthesis and nonstructural protein functions. J Gen Virol. 2015; 96(9):2483–500. Epub 2015/07/27. doi: 10.1099/jgv.0.000249 PMID: 26219641.

40. Utt A, Quirin T, Saul S, Hellstrom K, Ahola T, Merits A. Versatile Trans-Replication Systems for Chikungunya Virus Allow Functional Analysis and Tagging of Every Replicase Protein. PLoS One. 2016; 11 (3):e0151616. doi: 10.1371/journal.pone.0151616 PMID: 26963103.

41. Merkling SH, van Rij RP. Beyond RNAi: antiviral defense strategies in Drosophila and mosquito. Journal of insect physiology. 2013; 59(2):159–70. Epub 2012/07/25. doi: 10.1016/j.jinsphys.2012.07.004 PMID: 22824741.

42. Morazzani EM, Wiley MR, Murreddu MG, Adelman ZN, Myles KM. Production of virus-derived ping-pong-dependent piRNA-like small RNAs in the mosquito soma. PLoS Pathog. 2012; 8(1):e1002470. doi: 10.1371/journal.ppat.1002470 PMID: 22241995; PubMed Central PMCID: PMC3252369.

43. Cenik ES, Fukunaga R, Lu G, Dutcher R, Wang Y, Tanaka Hall TM, et al. Phosphate and R2D2 restrict the substrate specificity of Dicer-2, an ATP-driven ribonuclease. Mol Cell. 2011; 42(2):172–84. doi: 10.1016/j.molcel.2011.03.002 PMID: 21419681; PubMed Central PMCID: PMC3115569.

44. Blagrove MS, Arias-Goeta C, Di Genua C, Failloux AB, Sinkins SP. A Wolbachia wMel transinfection in Aedes albopictus is not detrimental to host fitness and inhibits Chikungunya virus. PLoS Negl Trop Dis. 2013; 7(3):e2152. Epub 2013/04/05. doi: 10.1371/journal.pntd.0002152 PNTD-D-12-01495 [pii]. PMID: 23556030; PubMed Central PMCID: PMC3610642.

45. Mousson L, Martin E, Zouache K, Madec Y, Mavingui P, Failloux AB. Wolbachia modulates Chikungunya replication in Aedes albopictus. Mol Ecol. 2010; 19(9):1953–64. Epub 2010/03/30. doi: 10.1111/j.1365-294X.2010.04606.x [pii]. PMID: 20345686.

46. Lu P, Bian G, Pan X, Xi Z. Wolbachia induces density-dependent inhibition to dengue virus in mosquito cells. PLoS neglected tropical diseases. 2012; 6(7):e1754. Epub 2012/08/01. doi: 10.1371/journal.pntd.0001754 PMID: 22848774; PubMed Central PMCID: PMC3404113.

47. Strauss JH, Strauss EG. The alphaviruses: gene expression, replication, and evolution. Microbiol Rev. 1994; 58(3):491–562. PMID: 7968923; PubMed Central PMCID: PMC372977.

48. Mayoral JG, Etebari K, Hussain M, Khromykh AA, Asgari S. Wolbachia infection modifies the profile, shuttling and structure of microRNAs in a mosquito cell line. PLoS One. 2014; 9(4):e96107. doi: 10.1371/journal.pone.0096107 PMID: 24759922; PubMed Central PMCID: PMC3997519.

49. Hussain M, Lu G, Torres S, Edmonds JH, Kay BH, Khromykh AA, et al. Effect of Wolbachia on replication of West Nile virus in a mosquito cell line and adult mosquitoes. J Virol. 2013; 87(2):851–8. Epub 2012/11/02. doi: 10.1128/JVI.01837-12 [pii]. PMID: 23115298; PubMed Central PMCID: PMC3554047.

50. Spuul P, Balistreri G, Kaariainen L, Ahola T. Phosphatidylinositol 3-kinase-, actin-, and microtubule-dependent transport of Semliki Forest Virus replication complexes from the plasma membrane to modified lysosomes. J Virol. 2010; 84(15):7543–57. doi: 10.1128/JVI.00477-10 PMID: 20484502; PubMed Central PMCID: PMC2897599.

51. Wickersheim ML, Blumenstiel JP. Terminator oligo blocking efficiently eliminates rRNA from Drosophila small RNA sequencing libraries. Biotechniques. 2013; 55(5):269–72. doi: 10.2144/000114102 PMID: 24215643; PubMed Central PMCID: PMC4077022.

52. Ashe A, Belicard T, Le Pen J, Sarkies P, Frezal L, Lehrbach NJ, et al. A deletion polymorphism in the Caenorhabditis elegans RIG-I homolog disables viral RNA dicing and antiviral immunity. Elife. 2013; 2: e00994. doi: 10.7554/eLife.00994 PMID: 24137537; PubMed Central PMCID: PMC3793227.

53. Kozomara A, Griffiths-Jones S. miRBase: integrating microRNA annotation and deep-sequencing data. Nucleic Acids Res. 2011; 39(Database issue):D152–7. doi: 10.1093/nar/gkq1027 PMID: 21037258; PubMed Central PMCID: PMC3013655.

54. Anders S, Huber W. Differential expression analysis for sequence count data. Genome Biol. 2010; 11 (10):R106. doi: 10.1186/gb-2010-11-10-r106 PMID: 20979621; PubMed Central PMCID: PMC3218662.

55. Lohse M, Bolger AM, Nagel A, Fernie AR, Lunn JE, Stitt M, et al. RobiNA: a user-friendly, integrated software solution for RNA-Seq-based transcriptomics. Nucleic acids research. 2012; 40(Web Server issue):W622–7. doi: 10.1093/nar/gks540 PMID: 22684630; PubMed Central PMCID: PMC3394330.

56. Langmead B, Salzberg SL. Fast gapped-read alignment with Bowtie 2. Nature methods. 2012; 9 (4):357–9. doi: 10.1038/nmeth.1923 PMID: 22388286; PubMed Central PMCID: PMC3322381.

57. Kim D, Pertea G, Trapnell C, Pimentel H, Kelley R, Salzberg SL. TopHat2: accurate alignment of transcriptomes in the presence of insertions, deletions and gene fusions. Genome Biol. 2013; 14(4):R36. doi: 10.1186/gb-2013-14-4-r36 PMID: 23618408; PubMed Central PMCID: PMC4053844.

58. Anders S, McCarthy DJ, Chen Y, Okoniewski M, Smyth GK, Huber W, et al. Count-based differential expression analysis of RNA sequencing data using R and Bioconductor. Nat Protoc. 2013; 8(9):1765–86. doi: 10.1038/nprot.2013.099 PMID: 23975260.

59. Robinson MD, McCarthy DJ, Smyth GK. edgeR: a Bioconductor package for differential expression analysis of digital gene expression data. Bioinformatics. 2010; 26(1):139–40. doi: 10.1093/bioinformatics/btp616 PMID: 19910308; PubMed Central PMCID: PMC2796818.
